# Splicing factor TRA2B enhances synthesis of androgen receptor variant AR-V7 in prostate cancer cells

**DOI:** 10.1172/JCI198264

**Published:** 2026-04-01

**Authors:** Nicholas Brittain, Alec Paschalis, Ryan Nelson, Beth Adamson, Laura Walker, Ruaridh Duncan, Graham R. Smith, Suzanne McGill, Richard J.S. Burchmore, Denisa Bogdan, Juan M. Jiménez-Vacas, Jonathan Welti, Wei Yuan, Craig N. Robson, Pasquale Rescigno, Sara Luzzi, Adam Sharp, Johann de Bono, Luke Gaughan

**Affiliations:** 1 Newcastle University Centre for Cancer, Paul O’Gorman Building, Framlington Place, Newcastle upon Tyne, United Kingdom.; 2 The Institute for Cancer Research, London, United Kingdom.; 3 The Royal Marsden NHS Foundation Trust, London, United Kingdom.; 4 Newcastle University Bioinformatics Service Unit, Medical School, Newcastle University, Newcastle, United Kingdom.; 5 Glasgow Polyomics, Wolfson Wohl Cancer Research Centre, College of Medical, Veterinary & Life Sciences, University of Glasgow, Glasgow, United Kingdom.

**Keywords:** Clinical Research, Oncology, Prostate cancer

## Abstract

Treatment of locally advanced and metastatic prostate cancer (PC) with androgen receptor–targeting (AR-targeting) therapies has limited durability, with disease eventually progressing to castrate-resistant PC (CRPC). Constitutively active AR splice variants (AR-Vs), such as AR-V7, play a key role in driving treatment resistance and disease progression. Importantly, the failure to attenuate AR-V function represents a major unmet clinical need, and as such, defining how AR-Vs are generated is likely to yield new therapeutic targets. Our knowledge of factors that mediate splicing of AR-V–encoding mRNAs remains limited. Here, we have employed an RNA-targeting CasRx approach to identify selective protein interactors of *AR-V7* mRNA in PC. TRA2B and its ortholog, TRA2A, were identified as splicing regulators of *AR* transcripts that facilitate AR-V synthesis at the expense of full-length AR isoforms. TRA2B expression correlated with *AR-V7* transcript in CRPC and attenuation of TRA2-mediated splicing diminished PC cell growth. Exploiting TRA2B function may therefore provide new therapeutic opportunities in advanced disease.

## Abstract 2

AR-Vs enable prostate cancers to progress during hormone therapy. This study has identified the protein TRA2B as being key for the synthesis of AR-Vs.

## Graphical abstract

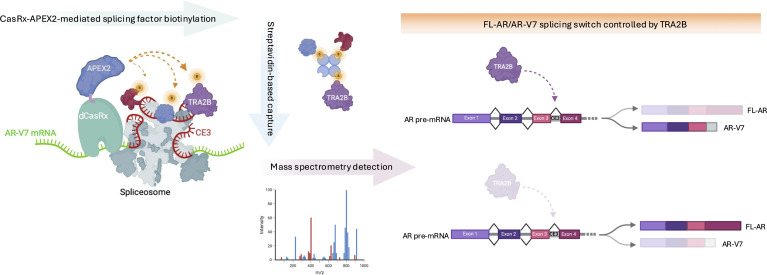

## Introduction

PC is the most prevalent male cancer worldwide with upwards of 1.3 million cases reported each year. AR signaling is a key disease driver and, as such, the mainstay for treating locally advanced and metastatic disease is through AR pathway blockade using androgen deprivation therapy (ADT) and/or antiandrogens, such as bicalutamide and enzalutamide (1–3). Unfortunately, not all patients show durable responses to these treatments; upwards of 80% develop resistance and succumb to disease progression to more aggressive castrate resistant PC (CRPC) (4–6). Importantly, reactivation of AR signaling occurs in this advanced stage of disease, in part through amplification and gain-of-function mutations of the *AR* gene and generation of alternatively spliced forms of the AR, termed AR-Vs (1, 7–9).

AR-Vs, including the clinically relevant isoform AR-V7, are undetectable in hormone-naive PC but emerge in approximately 75% of ADT-treated patients. By conferring resistance to both ADT and antiandrogens, AR-Vs significantly diminish overall survival of patients with advanced PC (10–12). In contrast with full-length AR (FL-AR), AR-Vs lack the C-terminal ligand-binding domain (LBD) but retain the transcriptionally active N-terminal transactivation domain (NTD) and DNA-binding domain (DBD) (13, 14); hence, AR-Vs remain active in both castrate levels of androgens and in the presence of antiandrogens and ADT (9, 12, 15). Difficulties selectively targeting the unstructured NTD and the highly conserved DBD have so far prevented effective AR-V blockade in the clinical setting (16, 17). An alternative approach to inactivate AR-V signaling is to identify and therapeutically exploit splicing factors that are critical for AR-V generation (18–20). Key to this is to enhance our understanding of aberrant *AR* pre-mRNA processing, which, in the case of *AR-V7*, favors splicing of cryptic exon 3 (CE3) to NTD- and DBD-encoding exons 1–3, at the expense of LBD-encoding exons 4–8. While several candidate-based studies have identified splicing and epigenetic regulators important for the synthesis of AR-Vs, including RBMX (21), SF3B2 (22), JMJD6 (23), and CWC22 (24), a comprehensive snapshot of splicing requirements for creating pathogenic *AR* transcripts remains lacking. Utilizing novel CRISPR-based approaches to study the *AR-V7* mRNA interactome may fill a key knowledge gap and provide the field with new therapeutic targets to attenuate AR-V synthesis.

To that end, we have utilized a strategy incorporating a catalytically inactive RNA-binding CasRx protein fused to the peroxidase enzyme APEX2 to enable detection of splicing factors that are directly bound to *AR-V7* transcripts to provide the first *AR-V7* transcript-specific interactome to date. Critically, we detected several splicing regulators that potentially interact with *AR-V7* transcripts, including the SR protein TRA2B. Depletion of TRA2B, and its paralog TRA2A, diminishes *AR-V7* mRNA and protein abundance and expression of canonical AR target genes in castrate conditions leading to compromised growth of AR-V7–positive PC. From a clinical perspective, *TRA2B* transcripts correlate with *AR-V7* mRNA in patient samples and are elevated in response to ADT and enzalutamide treatment, which is consistent with reduced overall survival. Moreover, morpholino-mediated blockade of TRA2B engagement with *AR* pre-mRNA compromises AR-V7 synthesis and suggests that antisense RNA technology exploiting TRA2B-RNA interactions may have clinical benefit.

## Results

### Development of dCasRx-APEX2 RNA binding pipeline.

**Figure 1:**
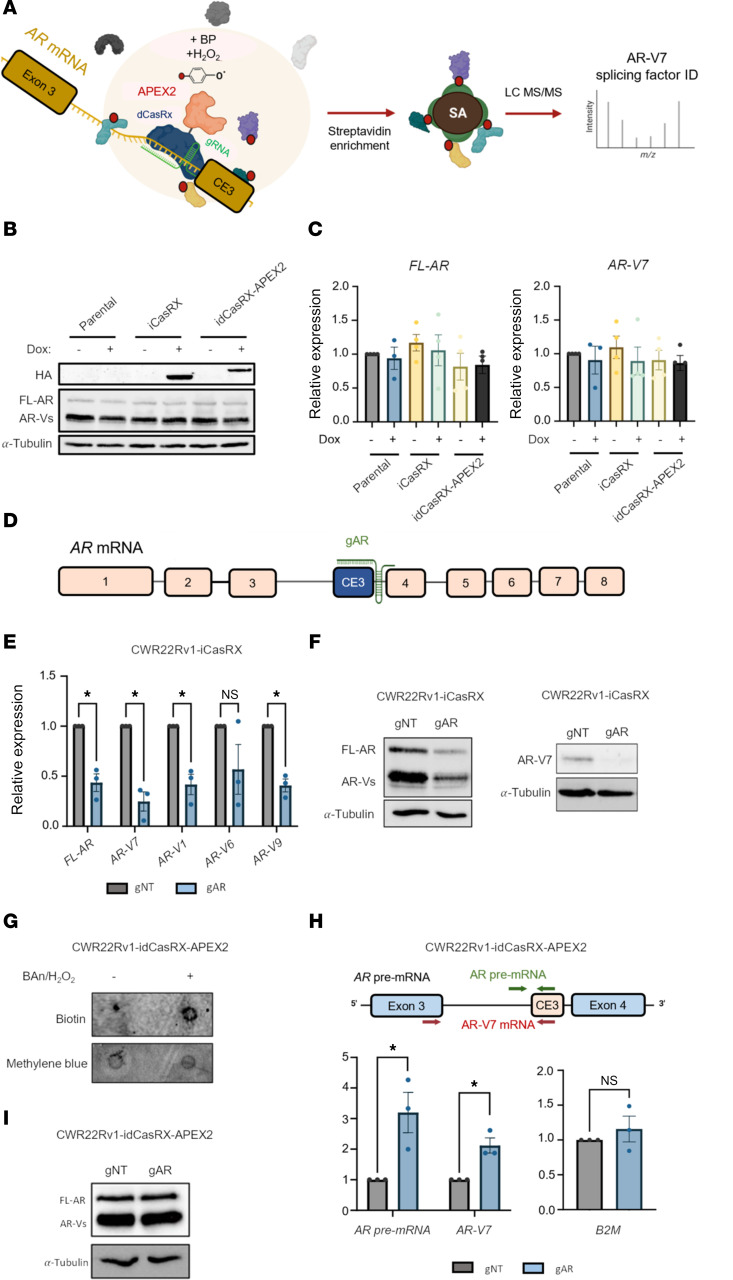
Validating selective dCasRX binding to AR-V7 mRNA. (**A**) Diagrammatic representation of the strategy to detect splicing regulators of pathogenic AR-Vs. When positioned at CE3 with a relevant gRNA, dCasRx-APEX2 can biotinylate proximal proteins for subsequent enrichment with magnetic streptavidin beads and protein identification by mass spectrometry. Stable doxycycline-inducible CWR22Rv1 derivatives expressing HA-CasRx (CWR22Rv1-iCasRX), an HA-dCasRx-APEX2 fusion (CWR22Rv1-idCasRX-APEX2), or parental control were treated with 1 μg/ml doxycycline for 72 hours prior to (**B**) Western analysis using anti-HA, -AR, and -α-tubulin antibodies or (**C**) RT-qPCR to assess relative abundance of FL-AR and AR-V7 transcripts. RT**-**qPCR data comprise *n* = 3 independent biological replicates, plotted as mean ± SEM. (**D**) Diagrammatic representation of the AR gene with position of AR CE3-targeting AR guide RNA (gAR) indicated. (**E**) CWR22Rv1-iCasRX were transfected with 25 nM of either a nontargeting gRNA (gNT) or gAR and induced with 1 μg/ml doxycycline for 72 hours before RNA was extracted for RT-qPCR and levels of FL-AR and AR-V7, -V1, -V6, and –V9 mRNAs were assessed. A 2-tailed unpaired *t* test was used for determination of statistical significance (*P < 0.05). (**F**) The experimental setup in **E** was repeated, and protein levels of AR-FL, AR-Vs, and AR-V7 were analyzed. α-tubulin was used as a loading control. Western blot is representative of *n* = 3 independent biological replicates. (**G**) CWR22Rv1-idCasRx-APEX2 were transfected with either gNT or gAR gRNAs and induced with doxycycline for 72 hours before incubation with biotin analine (BAn) and H_2_O_2_ for 2 hours and 2 minutes, respectively. Total RNA was extracted followed by streptavidin enrichment of total RNA extracts. A negative control induced with doxycycline but untreated with BAn/H_2_O_2_ was also included. RNA dot blot was performed using streptavidin-HRP. Methylene blue stain was used to confirm presence of RNA in each sample. (**H**) CWR22Rv1-idCasRx-APEX2 were transfected with either gNT or gAR gRNAs and induced with doxycycline for 72 hours before incubation with biotin analine (BAn) and H_2_O_2_ for 2 hours and 2 minutes, respectively. Total RNA was extracted and an RNA pulldown assay was performed with streptavidin prior to RT-qPCR to quantify levels of prespliced and postspliced CE3 mRNA enrichment between gAR and gNT-transfected samples. Data comprise *n* = 3 independent biological replicates, plotted as mean ± SEM. A 2-tailed unpaired *t* test was used for determination of statistical significance (*P < 0.05). (**I**) Western blot depicting levels of AR-FL and AR-V protein in CW22Rv1-idCasRx-APEX2 induced and transfected with the same gRNAs as in **H**. α-tubulin was used as a loading control.

We undertook an mRNA-protein capture approach, exploiting the selective RNA-binding and proximal protein biotinylation capacity of a dCasRx-APEX2 fusion, to identify proteins that selectively interact with and potentially regulate the inclusion of AR-V7–encoding CE3 into mature *AR*-V7 transcripts (Figure 1A and Supplemental Figure 1A; supplemental material available online with this article; https://doi.org/10.1172/JCI198264DS1). CasRx contains 2 higher eukaryote and prokaryote nucleotide-binding (HEPN) domains responsible for single-stranded RNase activity when activated by gRNA complementary with a target RNA (25–27). This mechanism has been applied in eukaryotic cells for potent, specific RNA knockdown analogous to siRNA approaches (28–31). Crucially, targeted mutation of the HEPN domains to create a catalytically dead CasRx variant (dCasRx), which retains RNA-binding capability without subsequent transcript cleavage, has been exploited to detect transcript-specific RNA-protein interactions (32–35). To that end, 2 stable CWR22Rv1 cell line derivatives expressing either WT CasRx (CWR22Rv1-iCasRx) or the dCasRx-APEX2 fusion (CWR22Rv1-idCasRx-APEX2) under the control of 1 μg/ml doxycycline were first created. Reassuringly, for the purpose of our study, steady-state levels of FL-AR and AR-V7 transcript and protein were not impacted by overexpression of either CasRx or dCasRx-APEX2 (Figure 1, B and C). We next designed and tested gRNAs to enable selective recruitment of dCasRx-APEX2 to the CE3 region of *AR* transcripts by using the RNA cleavage activity of enzymatically active CasRx as a surrogate for selective mRNA binding. We reasoned that successful downregulation of CE3-containing *AR* mRNAs upon CE3-targeting gRNA transfection in CWR22Rv1-iCasRx cells would evidence the suitability of our gRNAs to enable discriminate dCasRx-APEX2 binding to *AR* pre-mRNA and *AR-V7* transcripts. To that end, we validated both uptake of fluorescently labeled gRNAs into CWR22Rv1-iCasRx cell gRNAs (Supplemental Figure 1B) and CasRx-mediated transcript degradation using preoptimized p53-targeting gRNAs (gp53 1–3) in HEK293 cells (Supplemental Figure 1C) (32), indicating that our CasRx pipeline was functional. Crucially, transfection of CWR22Rv1-iCasRx cells with 25 nM of a CE3-targeting gRNA (gAR), which anneals to the 5’-end of CE3 (Figure 1D), but not a nontargeting gRNA (gNT), reduced transcript and protein abundance of multiple *AR-Vs*, including *AR-V7* and *FL-AR* over a period of 24–72 hours (Figure 1, E and F, and Supplemental Figure 2, A and B), indicating that our CE3-binding gRNA enables effective CasRx engagement with *AR-V7* mRNA and *AR* pre-mRNA.

To further support this, we applied an RNA biotinylation approach, which involves activating the APEX2 peroxidase activity of the dCasRx-APEX2-gAR complex by H_2_O_2_ treatment in the presence of biotin aniline (BAn), which preferentially labels RNA molecules (Supplemental Figure 2C) (36, 37). As such, successful recruitment of the dCasRx-APEX2-gAR complex to *AR* pre- and *AR-V7* mRNAs is expected to catalyze selective APEX2-mediated biotinylation of these transcripts, which can then be detected by combined streptavidin-based purification and RT-qPCR approaches. As shown in Figure 1G, biotinylated RNA abundance is increased in CWR22Rv1-idCasRx-APEX2–expressing cells subject to H_2_O_2_ and BAn treatment, and biotinylated CE3-containing *AR* pre-mRNA and *AR-V7* mRNAs but not a control B2M transcript (Figure 1H) were selectively captured by streptavidin-based immunoprecipitation, indicating engagement of the CasRx fusion with targeted *AR* transcripts. Reassuringly, FL-AR and AR-V protein levels were unaffected by expression of the dCasRx-APEX2-gAR complex (Figure 1I). Activation of APEX2-mediated protein biotinylation in our CWR22Rv1-idCasRx-APEX2 cells catalyzed selective biotinylation of nuclear proteins (Supplemental Figure 3, A–C), which were subsequently enriched by streptavidin-based immunoprecipitation. Overall, by validating selective dCasRx-APEX RNA binding and concurrent protein biotinylation, our experimental pipeline was optimized to detect splicing regulators of *AR-V7* transcripts.

### Defining the CE3-containing transcript interactome using dCasRx-APEX2.

**Figure 2:**
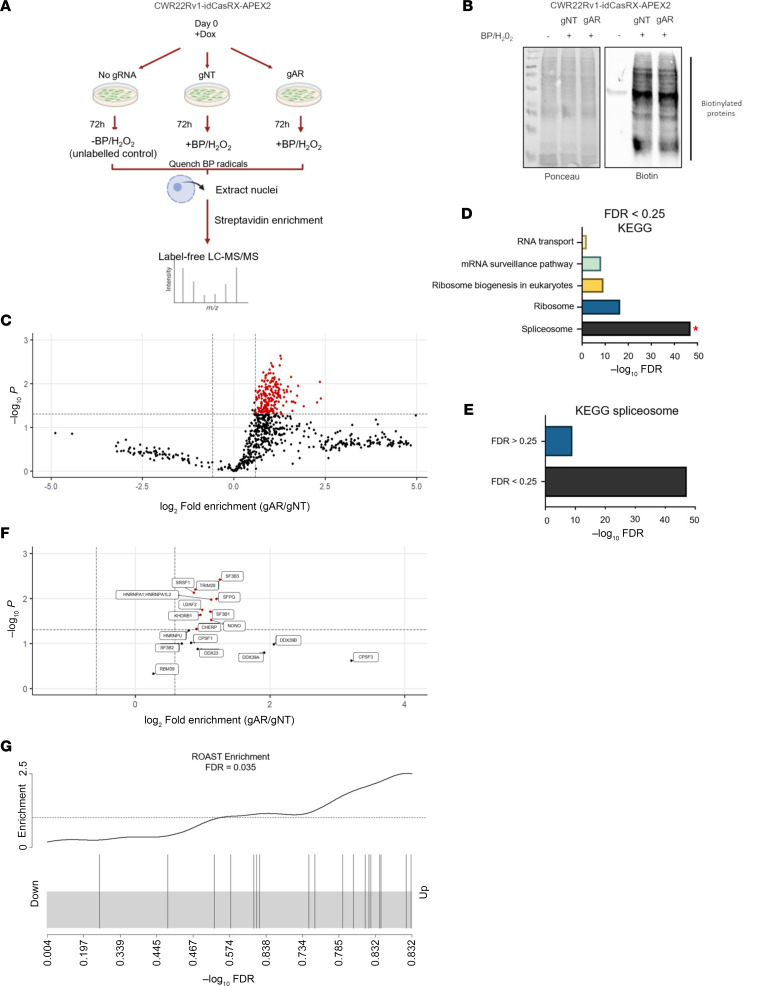
Positional proximal biotinylation at CE3 identifies numerous characterized AR-V7 splicing factors. (**A**) Summary of the label-free proteomics workflow used to detect splicing regulators involved in AR-V7 synthesis. CWR22Rv1-idCasRX-APEX2 were transfected with either nontargeting gRNA (gNT), AR gRNA (gAR), or no gRNA in the presence of 1 mg/μl doxycycline for 72 hours prior to biotin phenol (BP)/H_2_O_2_ treatment, streptavidin-based protein enrichment, and subsequent detection by mass spectrometry. (**B**) After applying the experimental setup detailed in **A**, a portion of extracted nuclear protein was retained for Western blot for biotinylated proteins to confirm APEX2 labeling compared with the unlabeled (-BP/H_2_O_2_). Ponceau was used to confirm equal protein loading. (**C**) Volcano plot of *limma-*analyzed protein iBAQ (derived from MaxQuant) value enrichment between gAR and gNT arms. Protein enrichment was calculated between AR g2 and NT gRNA arms using iBAQ values with the *limma* package. Enrichment cutoffs for visualization are *limma P* value (unadjusted) < 0.05 and linear fold enrichment > 1.5 (log_2_ 0.585). (**D**) KEGG analysis, performed using STRING, was applied to proteins enriched by gAR at a *limma* FDR < 0.25. The top 5 most significant KEGG terms are displayed and ranked by –log_10_ STRING FDR. KEGG spliceosome is highlighted by a red asterisk. (**E**) KEGG spliceosome –log_10_ STRING FDR comparison between high- (FDR < 0.25) and low- (FDR > 0.25) confidence CE3 interactors. (**F**) Protein enrichment results as in **C** were filtered for previously published AR-V7 regulators and are shown in the filtered plot. (**G**) ROAST was performed using the curated list of AR-V7 splicing factors as input (Supplemental Table 4). FDR represents ROAST-calculated significance of statistical enrichment for this list. Barcode plot represents ranking of –log_10_ FDR values for this list among all proteins from *limma* analysis.

To expedite splicing factor identification, CWR22Rv1-idCasRx-APEX2 transfected with either gNT or gAR were subject to proximal protein biotinylation, and streptavidin-based enrichment, as outlined in Figure 2A. An additional experimental arm, utilizing mock transfected cells and lacking APEX2 activation, was used to control for detection of endogenously biotinylated proteins. Successful in-cell protein biotinylation in both dCasRx-APEX2-gNT and -gAR experimental arms was validated prior to mass spectrometry analysis (Figure 2B). Utilizing an optimized mass spectrometry analysis workflow we have published previously (21), in which intensity-based absolute quantification (IBAQ) values were calculated for each protein, we sought to identify proteins demonstrating significant enrichment in our gAR-transfected experimental arm, which we classify as CE3-interacting proteins, over those detected in the control gNT samples. Critically, peptide quantities from each sample were normalized prior to MS/MS analysis from 4 independent experiments, which resulted in a greater degree of parity in our Log2-transformed iBAQ intensities between the gNT and gAR samples (Supplemental Figure 4) and, in doing so, would provide greater confidence in detecting proteins selectively binding CE3. As shown in Figure 2C, 669 proteins demonstrated differential enrichment in the dCasRx-APEX2-gAR experimental arm compared with the dCasRx-APEX2-gNT control, with 203 demonstrating a significantly enhanced enrichment, as determined using *limma* analysis of protein IBAQ values (*P* value, unadjusted, < 0.05 and linear fold enrichment > 1.5; Supplemental Table 4). Encouragingly, by separating our total enriched proteins into 2 categories representing false discovery rates (FDRs) of either less than 0.25 (high-confidence CE3 interactors) or greater than 0.25 (low-confidence CE3 interactors), we found a substantially greater representation of proteins related to the Kyoto Encyclopedia of Genes and Genomes (KEGG) spliceosome in the less than 0.25 group using STRING analysis (Figure 2, D and E, and Supplemental Table 3). Further analysis of our high-confidence CE3 protein interactors identified significant functional clustering related to mRNA processing, including 3′-end processing, while the low-confidence CE3 interactors (FDR > 0.25) were more associated with ribosomal biogenesis (Supplemental Figure 4B). These results likely reflect the expected functional enrichment of proteins seen upon dCasRx-APEX2-gAR complex targeting to an actively spliced 3′ terminal exon, such as CE3.

We next cross-referenced our full list of identified proteins from dCasRx-APEX2-gNT and -gAR experimental arms with those previously found to control *AR-V7* splicing (Supplemental Table 5). Importantly, we identified a considerable number of our significantly enriched CE3-interacting proteins have established roles in *AR-V7* splicing regulation, including SF3B1-3 and SFPQ (Figure 2F) (22, 38–40). Subsequent rotation gene set testing (ROAST) analysis was conducted (41), in which enrichment values of each published *AR-V7* splicing regulator detected in our assay were presented as a barcode plot, representing FDR, and subject to statistical significance testing against all detected proteins in our list, similar to gene set enrichment analysis (GSEA). Crucially, ROAST demonstrated that published *AR-V7* regulators have a statistically significant distribution toward our high-confidence CE3 interactors in our dataset (FDR = 0.035) (Figure 2G), supporting the use of our contemporary approach to successfully identify proteins that are potentially involved in *AR-V7* splicing.

### Cross-referencing high-confidence CE3 interactors with clinical data identifies TRA2B as a potential AR-V7 splicing regulator.

**Figure 3:**
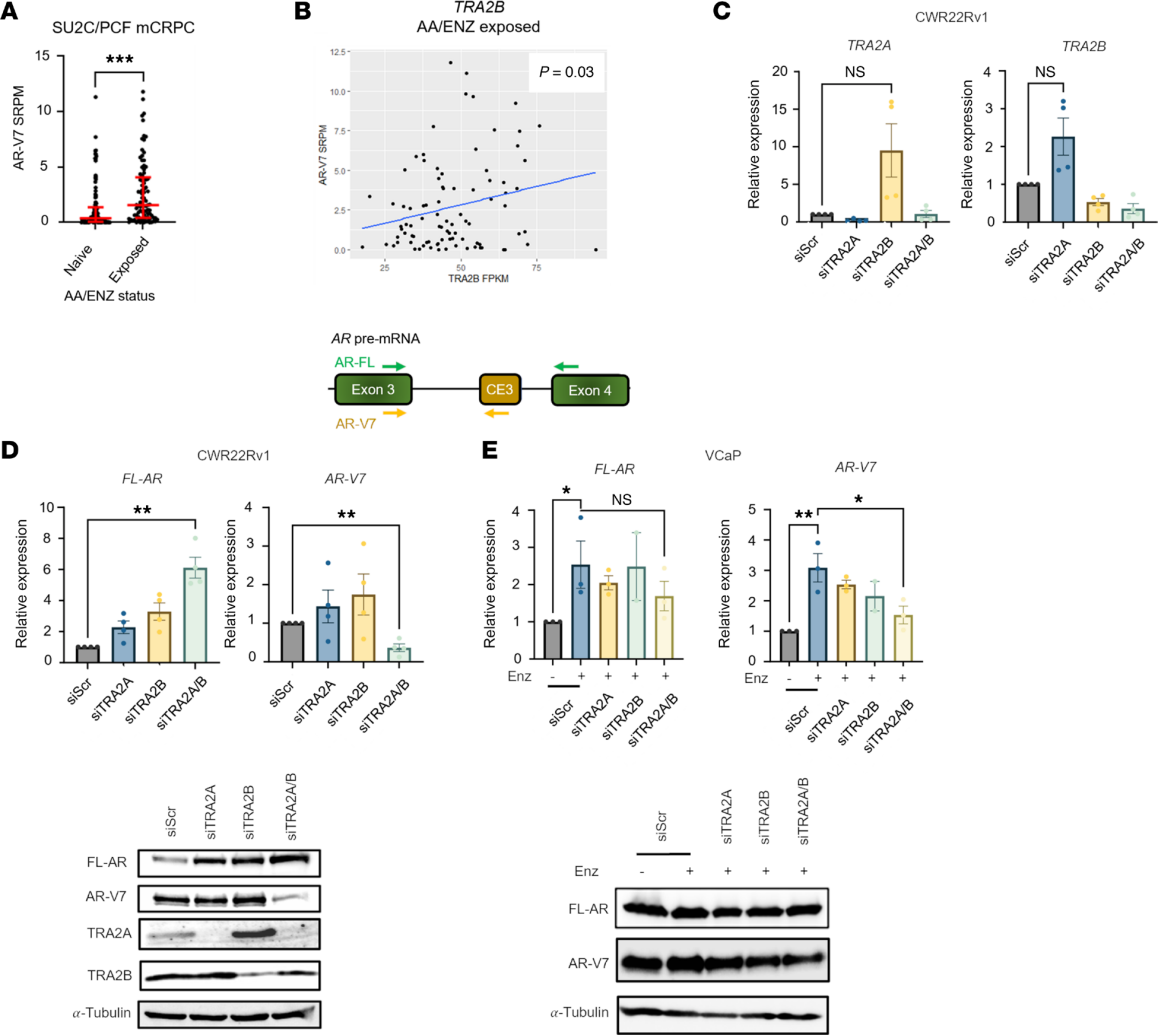
TRA2B and its paralog TRA2A regulate AR-V7 synthesis in PC. (**A**) Mean AR-V7 SRPM was compared between AA/ENZ naive and exposed patients from the SU2C/PCF mCRPC cohort (42). AA/ENZ naive comprised *n* = 106 patients, AA/ENZ exposed comprised *n* = 89 patients. Individual patient AR-V7 SRPM values are plotted. Significance was determined by 2-tailed unpaired *t* test (***P < 0.001). (**B**) *TRA2B* FPKM expression was correlated with AR-V7 SRPM in AA/ENZ naive and exposed patients. (**C**) CWR22Rv1 were transfected with 25 nM siRNA targeting TRA2A (siTRA2A), TRA2B (siTRA2B), or both (siTRA2A/B) for 72 hours before RT-qPCR and Western analysis of TRA2A and TRA2B transcript and protein levels, respectively. (**D**) Samples described in **C** were subsequently assessed for *FL-AR* and *AR-V7* transcript and protein. (**E**) VCaP cells grown in serum-containing media were transfected with TRA2A and TRA2B siRNA and treated ± 10 μM enzalutamide (Enz) for 72 hours before RT-qPCR and Western analysis of *AR-FL* and *AR-V7* transcript and protein levels. All qPCR data comprise *n* = 3 independent biological replicates, plotted as mean ± SEM, with a 1-way ANOVA used for determination of statistical significance (*P < 0.05, **P < 0.01).

Given the wide range of functional annotations associated with our 203 significant CE3-interacting proteins, it was important to refine the list to only those with functional and clinical relevance to the formation of *AR-V7* in advanced PC. We first chose to select only those proteins annotated to be directly involved in splicing (Supplemental Figure 5A), of which 63 fulfilled this criteria and were taken forward. We next hypothesized that expression of splicing factor genes that correlated with *AR-V7* transcript abundance in patients with advanced CRPC (*n* = 208), contained within the SU2C/Prostate Cancer Foundation (PCF) dataset (42), may be involved in *AR-V7* synthesis. Of the 63 CE3-interacting protein candidates, 27 were shown to correlate with *AR-V7*, including U2AF2 and SFPQ, which have been previously implicated in *AR-V7* splicing (39) (Supplemental Figure 5B). Similarly, we also compared transcript abundance of the 63 splicing factors to 3 independent AR-V7 transcriptional signatures, derived from cell lines (termed *AR_V7_UP*) (43) and patient samples (11, 44), with the assumption that splicing factors demonstrating correlative expression with AR-V7-driven genes would further evidence a potential role in *AR-V7* generation. Using mean Pearson’s correlation analysis, in which splicing factor genes were ranked on the basis of mean correlation with *AR_V7_UP* genes (Supplemental Figure 5C) and AR-V7 signatures (Supplemental Figure 5, C and D) in the SU2C/PCF CRPC cases, the expression of several splicing factors strongly correlated with AR-V7 transcriptional activity in at least 2 of the 3 gene sets, including TRA2B and THRAP3. Surprisingly, THRAP3 depletion elevated AR-V7 transcript abundance (Supplemental Figure 5E), which ruled this target out of our validation pipeline. As such, TRA2B was taken forward based on its expression correlating with (a) AR-V7 activity scores in 2 studies (43, 44); (b) *AR-V7* transcript in the SU2C/PCF cohort; and (c) *AR-V7* expression in enzalutamide- and abiraterone-treated patients with CRPC in the SU2C/PCF dataset (Figure 3, A and B).

### TRA2B and its ortholog TRA2A regulate AR isoform abundance in PC.

Transformer 2 (TRA2) was first characterized as an alternative splicing regulator involved in sex determination in *Drosophila melanogaster*, before 2 mammalian homologs, TRA2A (TRA2α) and TRA2B (TRA2β), were discovered (45, 46). TRA2A and TRA2B share approximately 75% sequence homology and are members of the serine-arginine (SR) protein family of splicing factors, which recruit spliceosome components at nascent transcript splice sites (47). Structurally, both homologs have an RNA-recognition motif (RRM) flanked by N- and C-terminal arginine/serine rich (RS) domains responsible for protein-protein interactions (48).

Interestingly, TRA2 proteins typically bind exonic rather than intronic splicing enhancer elements (49, 50), which supports the detection of TRA2B at CE3 of the *AR-V7* transcript in our Cas13d proteomics pipeline. Given that *TRA2A* expression does not correlate with *AR-V7* transcript abundance (SU2C cohort) (Supplemental Figure 6), TRA2B may represent the more functionally dominant ortholog in relation to AR splicing regulation. Consistent with a previously reported feedback loop, in which downregulated *TRA2B* expression resulted in a reciprocal change in *TRA2A* mRNA abundance, we observed that individual depletion of either TRA2A and TRA2B upregulated transcript and protein levels of the alternative ortholog in CWR22Rv1 cells (Figure 3, C and D), but not in VCaP cells (Supplemental Figure 7A), suggesting this mode of regulation is not ubiquitous, but nonetheless an important consideration for assessing TRA2 protein function. Furthermore, although these proteins are not functionally redundant (51), evidence from breast cancer cell lines has indicated knockdown of both TRA2A and TRA2B was required for splicing changes across a number of genes, including *CHEK1*, *ATRX*, *GLYR1*, and *CEP95* (52), suggesting that for at least some target RNAs, TRA2 paralogs can compensate adequately. As such, we chose to study the effect of both individual and combined TRA2 protein knockdown on *AR* isoform mRNA abundance in PC cells, as the effects of knocking down just TRA2B alone could be masked by the reciprocal upregulation of TRA2A.

While single depletion of TRA2A or TRA2B proteins had no effect on *AR-V7*, dual knockdown reciprocally diminished AR-V7 and upregulated FL-AR mRNA and protein levels in CWR22Rv1 cells (Figure 3D), suggesting the existence of a splicing switch in this cell line. Although no change in FL-AR was observed upon dual TRA2A and TRA2B knockdown in VCaP cells, enzalutamide-induced AR-V7 overexpression was significantly reduced (Figure 3E). Furthermore, individual depletion of TRA2B and dual knockdown of both TRA2 orthologs resulted in a marked reduction to AR-V7 levels in VCaP cells grown in steroid-depleted media supplemented with enzalutamide (Supplemental Figure 7B), supporting a critical role of TRA2B, and, to a lesser extent TRA2A, in controlling *AR-V7* transcript levels in conditions reflecting CRPC.

### TRA2 proteins facilitate CE3 inclusion into mature AR-V7 transcripts.

**Figure 4:**
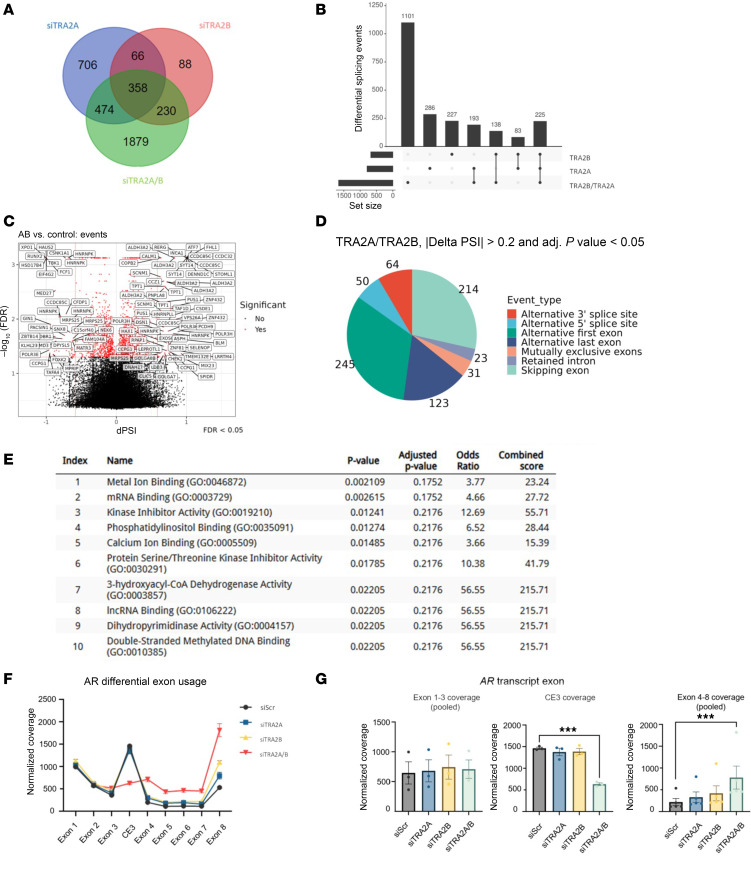
TRA2 proteins facilitate CE3 inclusion into mature AR-V transcripts. (**A**) RNA-seq was performed on CWR22Rv1 cells depleted of TRA2A, TRA2B, or both for 72 hours. Overlapping differentially expressed genes (FDR < 0.05 and linear fold change ± 1.5) for knockdown of TRA2A (siTRA2A), TRA2B (siTRA2B), or both (siTRA2A/B) versus scrambled control are shown in the Venn diagram. (**B**) Global splicing analysis was conducted using SUPPA2 (53) to assess altered splicing events between individual and dual TRA2 ortholog knockdown. Events that passed cutoffs of ΔPSI ± 0.6 and FDR < 0.05 were plotted as an upset plot. (**C**) Differential splicing events between control and dual TRA2A/B depletion that passed cutoffs of ΔPSI ± 0.6 and FDR < 0.05 are shown as volcano plots with significantly altered genes shown in red and annotated with corresponding IDs. (**D**) Global splicing patterns of CWR22Rv1 cells depleted of combined TRA2A/B knockdown were quantified by category (e.g., alternative first exon and skipping exon). Events that passed a significant Δ proportion spliced in (ΔPSI) value ± 0.2 (*P* < 0.05) were plotted as a pie chart including event quantification. (**E** and **F**) DEU analysis of RNA-seq BAM files was performed using *JunctionSeq*. Mean normalized exon counts for each siRNA treatment are plotted ±SEM for the AR exons. (**G**) Mean *JunctionSeq*-normalized exon counts for AR exons 1–3 and 4–8 were pooled. Mean normalized exon counts ± SEM are plotted across samples for the exons. Asterisks denote *JunctionSeq* FDR of DEU contrasts between indicated samples (***FDR < 0.00001). Not all samples have significance denoted for visualization ease.

To investigate more broadly the role of TRA2 proteins in transcription and splicing, RNA-seq was conducted in CWR22Rv1 cells depleted of TRA2A, TRA2B, or both. Initial quality control by principal component analysis (PCA) and hierarchical clustering demonstrated robust overlap within each experimental triplicate, with individual and dual TRA2A/B-depleted samples displaying largely distinct expression profiles (Supplemental Figure 8, A–C). Subsequent differential gene expression analysis across each experimental contrast revealed that dual depletion of TRA2 orthologs generated an appreciably greater number of differentially expressed genes (DEGs; *n* = 2,941) at a cutoff of FDR < 0.05 and linear fold change ± 1.5, compared with individual knockdown of TRA2A (*n* = 1,604) and TRA2B (*n* = 742) (Supplemental Figure 8D and Supplemental Tables 6–8). Interestingly, markedly fewer DEGs were detected in cells depleted of TRA2B compared with TRA2A, which could be a consequence of effective transcriptional buffering by augmented TRA2A expression in response to lowered TRA2B levels. Moreover, 706 genes were uniquely altered by lone TRA2A knockdown compared with just 88 for TRA2B, further potentiating a role for TRA2A-mediated transcriptional compensation in the absence of TRA2B (Figure 4A). Notably, 1,879 genes were uniquely differentially expressed by combined TRA2A/B knockdown, which was far in excess of unique DEG identities from individual ortholog knockdowns, suggesting TRA2 proteins likely regulate both unique and shared RNA targets.

Given the roles of TRA2A and TRA2B in RNA processing, we next performed global splicing analysis in cells depleted of individual and dual knockdown of TRA2 proteins using SUPPA (53) which calculates a ‘Δ proportion spliced in’ value that represents the quantity of alternatively spliced transcripts between experimental arms. Consistent with the more pronounced effect of dual TRA2A/B depletion on gene expression, we observed 1,101 significantly altered splicing events (*P* < 0.05) in response to combined TRA2 protein knockdown compared with 286 and 227 for individual TRA2A and TRA2B depletion, respectively (Figure 4, B and C, and Supplemental Figure 9, A and B). Interestingly, of the altered splicing events detected upon TRA2B knockdown, there was only a 37% overlap with those controlled by TRA2A, which again supports the notion of functional redundancy between the TRA2 orthologs. Next, by examining transcript composition, we found *Alternativ*e *first exon* and *exon skipping* as the most common changes to mRNA splicing in response to individual and dual TRA2 protein knockdown (Figure 4D and Supplemental Figure 9, C and D). Functional annotation of genes associated with the significant splicing alterations (Supplemental Tables 9–11) using Enrichr pathway analysis (54) identified several cellular processes impacted by individual and dual TRA2 ortholog depletion, including enrichment for RNA binding activity (Supplemental Figure 9, E–G).

To discover how TRA2 proteins control the fate of *AR/AR-V7* transcripts, we overlapped read alignment Binary Alignment Map (BAM) files with annotated exons to calculate normalized differential exon usage (DEU) across experimental arms using the *JunctionSeq* R Bioconductor package (55). Consistent with our earlier observations, *AR* transcript DEU was selectively altered by dual TRA2A/B knockdown in CWR22Rv1 cells in which inclusion of FL-AR-encoding exons 4–8 was significantly upregulated at the expense of AR-V7-encoding CE3 (Figure 4F). Crucially, mean coverage of constitutive exons 1–3, which encode the N-terminal transactivation and DNA-binding domains common in FL-AR and AR-V isoforms, was unchanged, supporting the concept that TRA2 proteins promote *AR-V7* splicing events to enhance *AR-V7* transcript synthesis without impacting de novo *AR* gene transcription or mature mRNA turnover (Figure 4G). Remarkably, DEU revealed that CE3 and exon 4 were among some of the most differentially utilized exons across the entire annotated transcriptome (Supplemental Figure 10A), with CE3 being the 11th most negatively differentially used exon upon TRA2A/B depletion. Furthermore, inclusion of CE1 and CE4, which encode the respective clinically relevant AR-Vs, AR-V1 and AR-V3 (56, 57), was also diminished by loss of TRA2 protein expression (Supplemental Figure 10B), supporting the concept that TRA2B and its paralog TRA2A are key splicing regulators of multiple AR-Vs in this model of CRPC.

**Figure 5:**
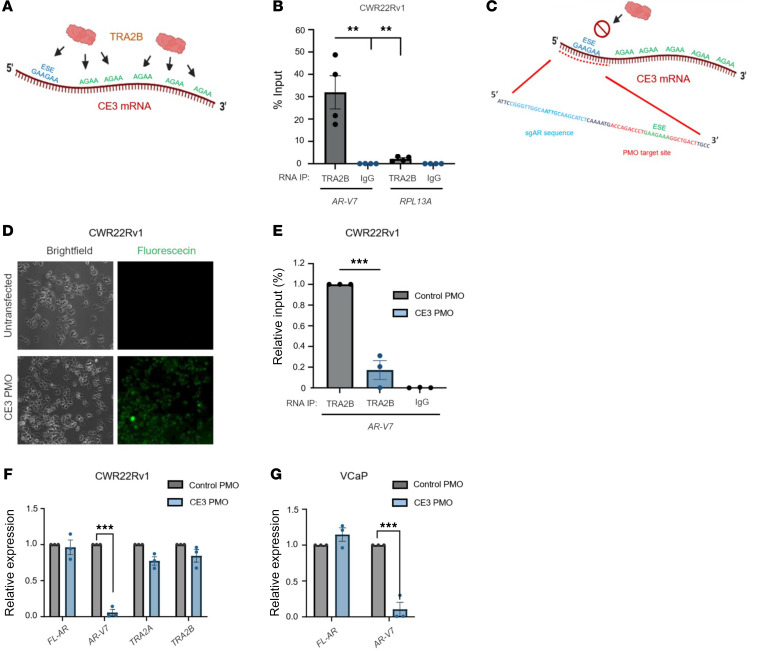
TRA2B binding to CE3 is required for AR-V7 synthesis. (**A**) Diagrammatic representation of TRA2B binding sites (AGAA) and the upstream exonic splicing enhancer (ESE) sequence in mRNA encompassing AR-V7-encoding CE3. (**B**) CWR22Rv1 cells were subject to RNA immunoprecipitation using either anti-TRA2B or control antibodies prior to qRT-PCR to quantify percentage input of TRA2B interaction with *AR-V7* and control *RPL13A* transcripts (*P < 0.05, **P < 0.01, and ***P < 0.001, as calculated using a 1-way ANOVA from at least 3 independent experiments). (**C**) Diagrammatic representation of TRA2B binding sites encompassing *AR-V7*-encoding CE3 and the phosphorodiamidate morpholino oligomers (PMOs) targeting a region encompassing the ESE of the *AR* pre-mRNA transcript (red dotted line). (**D**) 10× original magnification live-cell imaging of CWR22Rv1 cells transfected with and without 10 μM fluorescein-labeled CE3-targeting PMO for 48 hours to validate the transfection pipeline. (**E**) Cells transfected as in **D** with control or CE3-targeting PMOs were subject to RNA immunoprecipitation using either anti-TRA2B or control antibodies before RT-qPCR analysis to assess *AR-V7* transcript enrichment by TRA2B in the presence and absence of control or CE3-targeting PMOs (**P < 0.01 as calculated from 3 independent replicates using a 1-way ANOVA). CWR22Rv1 (**F**) and VCaP (**G**) were transfected with 10 μM CE3-targeting or control PMO for 48 hours prior to RT-qPCR analysis expression of the indicated mRNAs. qPCR data comprise *n* = 3 independent biological replicates, plotted as mean ± SEM and subject to a 1-way ANOVA (***P < 0.001).

Both TRA2 orthologs selectively bind RNA at consensus AGAA motifs. These theoretically occur once every 256 nucleotides (49, 53, 58), and an elevated tetramer frequency of greater than 1.5-fold has been previously used to select and validate TRA2 binding sites (59). Crucially, CE3 and the 3′-UTR of *AR-V7* mRNA contains an elevated AGAA frequency of 2.4-fold above average (Figure 5A). Moreover, TRA2 proteins bind exon splicing enhancer (ESE) elements composed of the hexameric GAAGAA sequence (60, 61), one of which is proximal to the sgAR-bound dCasRx-APEX2 binding site in CE3 and has been shown to be critical for *AR-V7* synthesis (62). Together, these observations further support the detection of TRA2B at the CE3 region of the *AR-V7* transcript in our proteomics pipeline. Consistent with this, RNA immunoprecipitation (RIP) using an anti-TRA2B antibody in CWR22Rv1 cells demonstrated significantly higher binding of TRA2B to *AR-V7* transcripts compared with control *RPL13A* mRNAs (Figure 5B). Interestingly, TRA2A was also found to interact with *AR-V7* mRNA (Supplemental Figure 11), albeit 20-fold less than TRA2B, which suggests that each ortholog has the capacity to control *AR-V7* splicing and likely explains the need to deplete both TRA2A and TRA2B proteins to downregulate *AR-V7* synthesis.

To define more specifically the site(s) of TRA2B interaction(s) on *AR-V7* mRNAs, we designed a fluorescein-labeled phosphorodiamidate morpholino oligomer (PMO) complementary to the 5′ region of CE3 mRNA, encompassing the GAAGAA-containing ESE (termed CE3 PMO), which we predicted to selectively engage TRA2B. As such, we hypothesized that the PMO would sterically hinder the binding of TRA2B to CE3-containing *AR-V7* mRNAs (Figure 5C). Upon transfection of the fluorescein-labeled CE3-targeting PMO (Figure 5D), we found a marked reduction in the interaction between TRA2B and *AR-V7* transcripts, which did not occur in the presence of the control PMO, suggesting that the GAAGAA-containing region of CE3 is a site of TRA2B engagement (Figure 5E). Importantly, compared with the scrambled PMO control, the CE3-targeting PMO selectively diminished *AR-V7* mRNAs in CWR22Rv1 and VCaP cells without impacting *FL-AR*, *TRA2* ortholog, or *AR-V5* and *AR-V6* expression (Figure 5, F and G, and Supplemental Figure 12, A and B), supporting the concept that TRA2 proteins facilitate selective splicing of *AR-V7* transcripts by engaging with the AGAA-rich CE3.

### TRA2 ortholog depletion diminishes AR-V signaling to impact PC growth.

**Figure 6:**
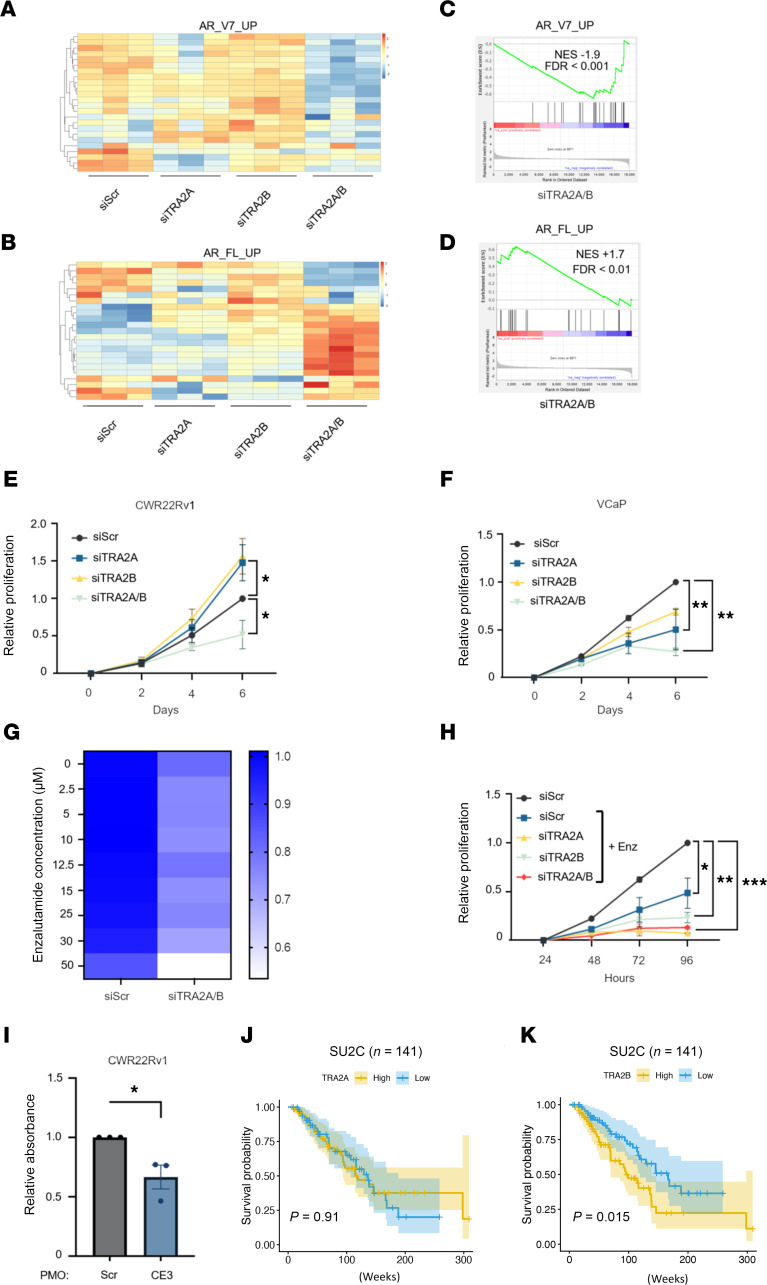
TRA2 depletion diminishes AR-V signaling and PC cell growth. Normalized RNA-seq counts were filtered for genes found in the AR_V7_UP (**A**) and AR_FL_UP (**B**) 25-gene signature (43). Heatmap displays z-score scaled normalized counts for each gene in this signature across each sample. Gene set enrichment analysis (GSEA) of AR_V7_UP (**C**) and AR_FL_UP (**D**) for the siTRA2A/B versus siScr experimental contrast. For heatmaps, red and blue indicate positive and negative z-scores, respectively. NES, normalized enrichment score for GSEA; FDR, GSEA false discovery rate. CWR22Rv1 (**E**) and VCaP (**F**) cells grown in serum-containing media were transfected with the indicated siRNAs, then treated with 0.1% DMSO, and proliferation was measured using SRB assays over the indicated time points. (**G**) CWR22Rv1 cells were treated and assayed as in **E** with addition of increasing doses of enzalutamide. Relative proliferation is plotted as a heatmap. (**H**) VCaP cells were transfected with the indicated siRNAs, treated with 0.1% DMSO or 10 μM enzalutamide, and proliferation was measured as in **G**. All SRB data were normalized to respective day 0 samples, before being scaled within each biological replicate to the NT/DMSO experimental arm. A 1-way ANOVA was used for determination of statistical significance between day 6 data points. Only results significant at α 0.05 or lower have significance denoted (*P < 0.05, **P < 0.01, ***P < 0.001). (**I**) CWR22Rv1 cells were transfected for 5 days with either scrambled (Scr) or CE3-targeting (CE3) phosphorodiamidate morpholino oligomers (PMOs) prior to SRB-based growth assays. Data are the mean of *N* = 3 experiments ± SD (*P < 0.05 as calculated using a 2-tailed paired *t* test). (**J** and **K**) Kaplan-Meier analysis (log-rank test) of OS in the SU2C/PCF mCRPC cohort according to TRA2A (**J**) and TRA2B (**K**) mRNA expression levels. High (yellow) and low (blue) groups were defined using the median expression level for each gene across all analyzed biopsies.

Our findings have provided evidence that siRNA-mediated TRA2 ortholog knockdown reprograms alternative splicing to diminish synthesis of clinically relevant *AR-V* transcripts and, for CWR22Rv1 cells, concurrently upregulates *FL-AR* mRNAs. It was therefore important to assess impact of dual TRA2A/B knockdown on AR signaling. Consistent with our observations of reciprocal alterations to AR isoform mRNAs in response to dual TRA2 ortholog knockdown, differential gene expression analysis incorporating 2 independent 25-gene expression signatures, *AR_V7_UP* and *AR_FL_UP* (43), demonstrated diminished AR-V7 signaling coincident with augmented FL-AR activity (Figure 6, A and B). Subsequent GSEA confirmed significant negative and positive enrichments in combined TRA2 protein knockdowns for *AR_V7_UP* and *AR_FL_UP*, respectively (Figure 6, C and D), as well as respective negative and positive enrichments for a 41-gene AR-V7 signature (44) and *Hallmark_AR response* (Supplemental Figure 13).

As a potential consequence of diminished AR signaling, CWR22Rv1 and VCaP cells demonstrated reduced growth in response to dual TRA2 protein depletion by approximately 50% and 70%, respectively (Figure 6, E and F). Interestingly, while individual TRA2A and TRA2B knockdown decreased growth of VCaP cells, the opposite was observed for CWR22Rv1 cells, which showed enhanced growth. Although unclear, the growth reduction in VCaP cells may be due to a modest reduction in AR-V7 levels (Figure 3E and Supplemental Figure 7B), while the upregulation of CWR22Rv1 growth could be attributed to reciprocal upregulation of TRA2 orthologs and increased FL-AR (Figure 3D). Interestingly, dual depletion of TRA2A and TRA2B in CWR22Rv1 enhanced the antiproliferative effect of enzalutamide (Figure 6G), while individual or dual TRA2A/B knockdown potentiated the effect of enzalutamide in VCaP cells (Figure 6H). Furthermore, using the selective PMO, which diminishes both TRA2B-CE3 interaction and *AR-V7* synthesis, we demonstrate a significant reduction in proliferation of CWR22Rv1 cells (Figure 6I). Finally, high *TRA2B* mRNA levels associate with shorter overall survival (OS) from CRPC biopsies in the SU2C/PCF (*P* = 0.015) and TCGA PRAD (*P* = 0.0067) cohorts, which is mirrored by TRA2A in the TCGA PRAD (*P* = 0.0072) cohort, but not in SU2C/PCF (*P* = 0.91) dataset (Figure 6, J and K, and Supplemental Figure 14, A and B), suggesting that aberrant AR splicing driven by upregulated TRA2 orthologs may lead to a poorer prognosis. Together, our findings suggest aberrant AR-V generation in advanced PC is driven, in part, by altered activity of TRA2 orthologs, and their blockade may offer a therapeutic opportunity to resensitize patients with CRPC to FL-AR-targeting agents.

## Discussion

Recent advances in development of AR-targeting therapeutics has seen considerable improvement to treatment outcomes in the hormone-naive and castrate-resistant settings. However, resistance to these agents, as a consequence of AR-V generation by aberrant alternative splicing, represents a major clinical challenge (9, 11). Critically, the appearance of the most clinically abundant AR-V, AR-V7, in response to AR signaling blockade, has highlighted a dynamic splicing switch that favors synthesis of AR-V7 concurrent with FL-AR to support CRPC growth (11). While several candidate and genetic screening approaches have been utilized successfully to identify numerous splicing and epigenetic regulators important for *AR-V7* generation, such as RBMX (21), JMJD6 (23), and SF3B2 (22), there remains no direct and unbiased study of global protein interactors of CE3 mRNA.

To address this, we have developed a contemporary dCasRx-APEX2 approach to define, for the first time to our knowledge, the protein interactome of *AR* CE3 mRNA in a model of CRPC. Crucially, while a similar approach has been previously applied to identify RNA-protein interactions in HEK293 cells (63), our study is the first to our knowledge to assess pathogenic splicing in a cancer setting. We identified 203 selective, significantly enriched CE3-interacting proteins, providing a rich source of potential regulatory mediators dictating *AR-V7* splicing. Functional interrogation of the CE3 interactome demonstrated an overrepresentation of proteins involved in mRNA splicing, processing, and 3’-end maturation, all processes that would be predicted at an actively spliced 3’-terminal exon. The overlap between our CE3-interactome and the current knowledge base of published *AR-V7* splicing regulators, including SRSF3 (40) and DDX39A (64), further supported the utility of the dCasRX-APEX2 approach to detect regulators of *AR-V7* synthesis. Subsequent interrogation of the SU2C/PCF (42) patient cohort and AR-V7 activity signatures (44) identified TRA2B as one of several CE3-interacting proteins, demonstrating significant correlation with *AR-V7* expression and activity.

Mechanistically, depletion of TRA2B, and its ortholog TRA2A, dramatically impacted *AR* pre-mRNA splicing patterns in CWR22Rv1 cells, resulting in concurrent up- and downregulation of *FL-AR* and *AR-V7*, respectively, potentiating existence of an *AR* splicing switch governed by TRA2 proteins. The requirement to deplete both TRA2 orthologs in CWR22Rv1 cells to expedite *AR* splicing changes is consistent with the existence of a reciprocal feedback loop in which TRA2B facilitates inclusion of a poison exon into the TRA2A transcript, leading to its degradation (65). As such, TRA2A abundance is elevated upon TRA2B knockdown, which may enable it to control similar splicing activities to the more dominant ortholog TRA2B. This is evidenced by TRA2A regulating a high proportion of TRA2B-mediated splicing events and itself interacting with *AR-V7* transcripts, albeit less than TRA2B. The lower abundance of TRA2A may also help to explain why we failed to detect it in our mass spectrometry pipeline. This mode of regulation is also apparent in breast cancer cells in which depletion of both paralogs was required to elicit significant splicing changes in a range of transcripts, including CHEK1 (52). However, in enzalutamide-treated VCaP cells, individual TRA2 protein knockdown reduced *AR-V7* generation, albeit less than dual depletion, and there was no evidence of reciprocal TRA2 paralog upregulation after single TRA2 depletion. The reasons for this difference between PC cell lines is not yet clear. It is possible that posttranslational modifications provide an additional layer of TRA2 regulation in CRPC, as TRA2B is known to be phosphorylated by kinases including SRPK1 and CLK1, which modulate its activity (66, 67). Interestingly, examination of CWR22Rv1 RNA-seq experiments showed that *SRPK1* expression was significantly downregulated upon combined TRA2A/B depletion, which may potentiate the effects of knockdown by altering phosphorylation status of any remaining TRA2 protein. Therefore, comparing posttranslational modification status and the interactome of TRA2 orthologs between VCaP and CWR22Rv1 cells could allow a greater mechanistic insight into their regulation of AR-V7 splicing.

Evaluation of global splicing changes in cells depleted of individual and dual TRA2 proteins provided evidence that both proteins regulated many splicing events, with *alternative first exon* and *exon skipping* being the most predominant changes, which, as expected, was further pronounced upon depletion of both. Importantly, DEU analysis of normalized alignment coverage across AR exons indicated no measurable alteration to usage of *AR* exons 1–3 in response to TRA2A/B knockdown, implying rates of transcription and RNA turnover are unaffected by loss of the 2 splicing factors. Critically, however, a significant reduction in CE3 inclusion into mature mRNAs was observed upon dual TRA2A/B depletion, while utility of FL-AR–encoding exons 4–8 was markedly enhanced, which is consistent with TRA2 proteins regulating a splicing switch beyond exon 3. Furthermore, similar changes to exon usage were seen for AR CE1 and CE4, indicating TRA2 proteins may control splicing of multiple AR-Vs in addition to *AR-V7* in CRPC. Strikingly, DEU results showed that *AR* CE3 was the 11th most differentially negatively used exon upon combined TRA2 depletion across the entire annotated transcriptome, suggesting that TRA2 function is particularly relevant to *AR-V7* splicing. In support of this, transfection of a CE3-targeting PMO, which is complementary to the TRA2-binding GAAGAA ESE sequence at the 5’ end of CE3 and diminishes TRA2B interaction with *AR-V7* mRNA, resulted in potent and selective abrogation of *AR-V7* levels in both CWR22Rv1 and VCaP cells. Interestingly, we did not observe a parallel increase in *FL-AR* splicing upon PMO transfection in either PC cell line, alluding to mechanistic differences between steric blockade of the splicing enhancer and siRNA depletion of TRA2 orthologs.

Our finding that TRA2 ortholog knockdown diminished growth of PC cells and potentiated the effect of enzalutamide supports the role of TRA2 proteins in generating AR-Vs and diminishing response to AR-targeted agents. This is consistent with enhanced expression of TRA2B in both AR-V7–positive CRPC patients (SU2C) and patients with CRPC treated with abiraterone and enzalutamide (SU2C). From a translational standpoint, we have shown that elevated TRA2B expression reduces OS of patients with CRPC, suggesting that its involvement in AR-V7 synthesis potentially contributes to worse prognosis of advanced disease. In all, we have developed a robust splicing factor detection and validation pipeline that has shown that TRA2 proteins play a critical role in AR-V synthesis by modulating cellular splicing. TRA2B may represent the more functionally dominant and clinically relevant paralog in relation to AR splicing regulation, and so, drugging this alone may be sufficient to achieve the desired therapeutic benefit with fewer side effects. Additional work to develop small compound inhibitors/RNA-based therapeutics and validate the clinical utility of TRA2 protein inhibition in CRPC is now merited.

## Methods

### Sex as a biological variable.

Given that PC/CRPC is exclusively a male disease, all cell lines and human-derived PC/CRPC biopsies used in this study are of male origin.

### Cell lines and cell culture reagents.

CWR22Rv1 derivatives and VCaP cell lines were maintained in RPMI-1640 and DMEM, respectively, supplemented with 10% FBS (Thermo) and penicillin-streptomycin (Sigma). For steroid depletion experiments, FBS was substituted for dextran-coated charcoal stripped FBS (VWR S181F-500). Enzalutamide (Selleckchem S1250) was dissolved in DMSO (Sigma D5879) and used at a final concentration of 10 μM.

### RNA extraction, quantitative RT-PCR, and Western blotting.

RNA was isolated from cells using TRIzol Reagent (Life Sciences, Invitrogen) according to the manufacturer’s handbook as described in ref. 68. Resultant RNA was subject to cDNA synthesis using the M-MLV reverse transcriptase system (Promega). cDNA was diluted in 130 μL RNase/DNase-free water and analyzed by real-time quantitative PCR (RT-qPCR), incorporating SYBR Green DYE 1 (Life technologies) and custom primers purchased from Sigma-Aldrich (Supplemental Table 1), using a QuantStudio 7 Flex Real-Time PCR System (ThermoScience). Ct values were normalized to *RPL13A* using the ΔΔCT method. Western blotting was performed as before (69), and antibodies used in the study are listed in Supplemental Table 2.

### Plasmid construction and creation of CWR22Rv1-iCasRx/-iCasRx-APEX2 derivatives.

CasRx pXR001 (#109049) and catalytically inactive CasRx (dCasRx) pXR002 (#109050) expression plasmids were purchased from Addgene. dCasRx-APEX2 expression plasmid pXR002-APEX2 was created by digestion and ligation of a *Bam* HI 5′/3′ flanked gBlock (IDT) encoding the APEX2 ORF into the *Bam* HI site of pXR002, resulting in a dCasRx-APEX2 fusion.

pCasRx and pdCasRx-APEX2 mammalian expression vectors were purchased from VectorBuilder. pTLCV2-CasRx and pTLCV2-dCasRx-APEX2 were created by subcloning *Bsh* TI*/Bam* HI-digested CasRx and dCasRx-APEX2 ORFs from respective pCasRx and pdCasRx-APEX2 into the *Bsh* TI*/Bam* HI-digested pTLCV2 (Addgene #87360) recipient plasmid. Plasmid sequences were verified by Sanger sequencing (GENEWIZ). Resultant CWR22Rv1-iCasRx and CWR22Rv1-iCasRx-APEX2 derivatives were generated that inducibly express CasRx and dCasRx-APEX2, respectively, in response to 1 mg/ml doxycycline.

### Plasmid, siRNA, and, gRNA transfection.

Unless otherwise specified, all plasmids used throughout were reverse transfected at the amounts indicated with *Trans*IT-LT1 transfection reagent (MIR 2300) according to manufacturer instructions using a 3:1 (μl:μg) LT1:plasmid ratio in Opti-MEM I (Thermo 31985062). Where indicated, expression of eGFP was assessed by imaging cells with a TE2000 fluorescence microscope (Nikon Corporation). CWR22Rv1 derivatives and VCaP cells were reverse transfected in 6-well plates with siRNA (see Supplemental Table 3 for sequences) at a 25 nM final concentration using 0.2% lipofectamine RNAiMAX according to manufacturer instructions as in ref. 68. All CasRx gRNAs were designed using CasRxesign (available at https://cas13design.nygenome.org/) (70) and utilized as single custom RNA oligos (Sigma) comprising the CasRx gRNA 30 nt direct repeat (DR) sequence, 5′ - AACCCCUACCAACUGGUCGGGGUUUGAAAC - 3′, followed by a 22–23 nt spacer with complementarity to mRNA target sequence(s) (see Supplemental Table 3). At the point of transfection, expression of CasRx/dCasRx-APEX2 was induced using 1 μg/ml doxycycline. Culture media and doxycycline was refreshed after 48 hours and cells grown for a further 24 hours before harvest.

### Protein biotinylation and streptavidin immunoprecipitation.

HEK293FT or CWR22Rv1 cells expressing dCasRx-APEX2 were treated with 500 μM biotin-phenol for the timeframes indicated at 37°C and 5% CO_2_, before hydrogen peroxide (Sigma H1009) was added to a final concentration of 1 mM and plates were gently swirled by hand for the timeframes indicated at room temperature. Media was then quickly aspirated and replaced with APEX2 quenching buffer (100 mM sodium ascorbate (Sigma A7631), 10 mM TROLOX (Sigma 238813) and 10mM sodium azide (VWR 786-299) in PBS). APEX2 quenching buffer was removed and replaced 3 times for a total of 4 x quenching washes before immunoprecipitation of resultant biotinylated proteins as described in ref. 69. Streptavidin-bound protein samples in 100 μl 50 mM ammonium bicarbonate were prepared for LC-MS/MS (conducted by Polyomics) by tryptic digest and drying via a filter aided sample preparation (FASP) protocol. Briefly, samples were denatured in 4% (w/v) SDS, 100 mM Tris/HCl (PH 7.6), and 0.1 M DTT and mixed with UA (8 M urea [Sigma, U5128] in 0.1 M Tris/HCl [pH 8.5]). Samples were than alkylated using 0.05 M iodoacetamide in UA. Streptavidin-bound proteins were trypsin digested at 37°C overnight and then mixed with 10% acetonitrile, acidified in trifluoracetic acid, and dried down. Digested peptides were subsequently solubilized in 100 μl 100 mM triethylammonium bicarbonate (TEAB), and their concentration was determined using a DeNovix DS-11 spectrophotometer against a standard curve of HeLa cell digested peptide standards (Thermo 88328). 5 μg of each peptide sample was then dried down, before a 5 μl volume of solubilized peptide was desalted and concentrated on a trap column in 1% acetonitrile and 0.1% formic acid, and peptide separation was performed on a PepMap C18 reversed phase column using a formic acid/acetonitrile solvent gradient. LC eluates underwent electrospray ionization using Sharp Singularity emitters (Fossil Ion Tech), followed by peptide ion detection on an Orbitrap Elite mass spectrometer (Thermo).

Thermo Orbitrap RAW file outputs were provided, and MaxQuant (v2.0.3.0) (71) was used for peptide and protein-level intensity quantification of mass spectrometry outputs, with the inbuilt Andromeda search engine (72) run against a *Homo sapiens* Uniprot (Swiss-Prot and TrEMBL) FASTA proteome. MaxQuant was run using label-free intensity based absolute quantification (iBAQ) with default settings for instrument type ‘Orbitrap’ selected. Peptide-spectrum match and protein-level false-discovery rate thresholds were set at 1%, and the MaxQuant match between runs algorithm was enabled. Protein-level iBAQ values were subsequently read into R (v4.1.2, R Core Team, 2021) via the RStudio development environment (v2022.07.0, RStudio Team, 2022) for data processing. Contaminants, such as keratin, and reverse sequences were removed, and only proteins identified by greater than or equal to 2 unique peptides were retained.

Individual replicates were batch processed separately. First, iBAQ values in the corresponding unlabeled control sample were subtracted from iBAQ values in gNT and gAR samples, and any resulting negative values were set to 0. Proteins that had positive iBAQ values in at least 3 out of 4 replicates for either gRNA arm were retained for analysis. Values of 0 were imputed with half the minimum iBAQ value within that replicate. iBAQ values were then log_2_ transformed. For calculation of protein enrichment, a moderated *t* test with a paired design was implemented using the *limma* package (v3.50.3) (73) for comparison of log_2_ iBAQ values between AR g2 and NT gRNA arms. STRING (74) was used for functional analysis of proteins enriched at the specified significance thresholds. Rotation gene set testing (ROAST) and barcode plots were implemented using *limma*.

### RNA immunoprecipitation.

5 × 10^6^ CWR22Rv1 cells were subjected to RNA immunoprecipitation (RIP) according to the published protocol by Baker et al. (75), using 5 mg anti-TRA2A, anti-TRA2B, or isotype control antibodies. Immunoprecipitated complexes were subject to Trizol-based RNA extraction and cDNA synthesis as described (24). Enrichment of target RNA(s) between mRNA-targeting versus NT gRNA samples was compared by normalizing samples to their respective input and calculating relative fold enrichment as described below.

### RNA biotinylation and streptavidin-pulldown assay.

A total of 5 × 10^6^ CWR22Rv1-idCasRx-APEX2 cells were reverse transfected in 150 mm dishes with 25 nM of either gNT or gAR gRNAs using 0.2% RNAiMAX and expression of dCasRx-APEX2 was induced with doxycycline. After 72 hours, cells were incubated with 500 μM biotin-aniline for 2 hours at 37°C and 5% CO_2_, before H_2_O_2_ was added to a final concentration of 1 mM and plates were gently swirled by hand for 2 minutes at room temperature. Media was quickly aspirated and replaced with APEX2 quenching buffer. APEX2 quenching buffer was removed and replaced 3 times for a total of 4 quenching washes, which was followed by 4 PBS washes. Cells were subsequently lysed in an appropriate volume of TRIzol Reagent.

Total RNA was extracted using TRIzol according to manufacturer instructions. A total of 2 μg of RNA was taken and stored at –80°C for use as an input sample, while biotinylated RNA in the remaining sample was pulled down using Pierce streptavidin magnetic beads. Streptavidin beads and total RNA were used at a 1:2.5 (μl:μg) bead:RNA ratio. RNA was stored at –80°C while beads were washed and blocked. An appropriate volume of beads was washed 2 × 1 ml in ice-cold nuclease-free NT2 buffer and resuspended in 200 μl 0.1 M NaOH/0.05 M NaCl in nuclease-free H_2_O supplemented with 4% nuclease-free NT2 and incubated for 2 minutes at room temperature. Beads were then resuspended in 200 μl 0.1 M NaCl in nuclease-free H_2_O supplemented with 4% nuclease-free NT2 and incubated at room temperature for a further 2 minutes. Streptavidin beads were subsequently blocked by overnight rotation at 4°C in 500 μl RNA-blocking buffer (1 mg/ml BSA, 1 mg/ml yeast tRNA [Fisher 11508736] in nuclease-free H_2_O, supplemented with 4% nuclease-free NT2).

After streptavidin blocking, total RNA was thawed on ice. Beads were washed in 2 × 1 ml nuclease-free NT2 buffer and resuspended in 500 μl 0.05 M NaCl in nuclease-free water, and thawed RNA was added. Bead/RNA mixtures were rotated for 90 minutes at 4°C followed by 30 minutes at room temperature, before being washed 7 × 1 ml in RIPA buffer. Washed beads were resuspended in 100 μl digest/elute buffer (20 mM DTT, 5 mM biotin, 200 μg/100 μl proteinase K, 200 U/ml RNaseOUT, 0.2 M NaCl) and incubated for 1 hour at 42°C, followed by 1 hour at 55°C with regular vortexing. 1 ml TRIzol was then added to beads/buffer mixture, vortexed thoroughly, and RNA extracted. RNA was also extracted from input samples, with the final RNA resuspension being done in 15 μl nuclease-free water due to expected low yields. Input samples were thawed and equal amounts of input/enriched RNA were reverse transcribed and analyzed by qPCR. Target RNA enrichment between gRNA samples was calculated as below, with *RPL13A* used as a housekeeping gene and fold enrichment calculated using the equation: 2^–ΔΔCt^(AR g2) / 2^–ΔΔCt^(NT).

RNA biotinylation was confirmed by dot blot using 500 ng RNA spotted onto a BrightStar Plus positively charged nylon membrane (Thermo AM10102) and crosslinked for 2 × 30 seconds using a SpectroLinker XL-1000 UV Crosslinker (Spectronics Corporation), before being air dried for 15 minutes. The membrane was then blocked in PBS + 10% SDS and 1 mM EDTA for 20 minutes at room temperature, before incubation with 1:2,500 streptavidin HRP for 1 hour at room temperature. Membranes were then washed for 2 × 10 minutes in each of PBS + 10% SDS, PBS + 1% SDS, and PBS + 0.1% SDS, before chemiluminescence or total RNA using methylene blue stain (0.4 M acetic acid [Fisher A/0400/PB17], 0.4 M sodium acetate [Sigma S2889], and 0.2% [w/v] methylene blue [Sigma M9140]).

### Differential splicing analysis.

Transcript usage was estimated by pseudoaligning the RNA-seq data to version 109 of the human transcriptome obtained from Ensembl (“GRCh38.109”) using Salmon version 1.10.0. Differential splicing events were assessed using SUPPA2 version 2.3 (53), using the commands suppa.py generateEvents, suppa.py psiPerEvent and suppa.py diffSplice, as described in https://github.com/comprna/SUPPA/wiki/SUPPA2-tutorial Figures were generated with R version 4.2.2, using ggplot2_3.4.4, ggrepel_0.9.4 (for volcano plots), ggpubr_0.6.0 (for pie charts), and UpSetR_1.4.0 (for upset plots). Expression of TRA2 orthologs in the RMH patient cohort was conducted as described (69).

### Patients.

For the SU2C/PCF cohort, previously described whole exome and transcriptome mCRPC patient sequencing data with linked longitudinal clinical outcomes data was downloaded and reanalyzed (42).

### Statistics.

Unless otherwise stated, all graphical data represent the mean of 3 individual experiments and error bars indicate ±SEM. We conducted 2-tailed unpaired or paired *t* tests and 1-way ANOVA where appropriate (described in figure legends) using GraphPad Prism 8 software, and *P* < 0.05 was classified as statistically significant. All results were plotted using GraphPad Prism (v9.3.1) or R (v4.1.2, 2021) via the RStudio development environment (v2022.07.0, 2022). The tidyverse software suite (v2.0.0) was used for data processing and plotting in R throughout.

### Study approval.

Approval for patient involvement in this study was granted by the Royal Marsden Hospital Ethics Review committee (reference no. 04/Q0801/60) as described in ref. 77.

### Data availability.

A Supporting Data Values file including values underlying graphed data and reported means presented in main and supplemental figures is provided. RNA-seq data generated in this study is publicly available with the accession number GSE311986.

## Author contributions

NB performed most of the experiments and, together with BA, developed the biotinylation workflow. LW, RN, RD, JMJV, AP, SL, JW, and LG performed some experiments. SM and RJSB provided guidance on biotinylation samples preparation and conducted all proteomics. NB, DB, WY, AP, and GRS performed all bioinformatics. CNR, PR, AS, JdB, and LG devised the project. NB, AP, AS, JdB, and LG wrote the paper.

## Funding support

- Prostate Cancer UK.
- The Movember Foundation through the London Movember Centre of Excellence (CEO13_2-002).
- The John Black Charitable Foundation and Prostate Cancer Foundation (18CHAL06 and 20YOUN17).
- Cancer Research UK (Centre Programme grant).
- Experimental Cancer Medicine Centre grant funding from Cancer Research UK and the Department of Health.
- Biomedical Research Centre funding to the Royal Marsden.
- The Wellcome Trust (AS).
- Cancer Research UK Newcastle Centre (C9380/A25138
- to NB).
- The Ken Bell Bursary and JGW Patterson Foundation
- (12/21 NU009331 to BA).
- Prostate Cancer Research (PCR-6955 to RN and LW).

## Supplementary Material

Supplemental data

Unedited blot and gel images

Supplemental tables 1-11

Supporting data values

## Version 1 04/01/2026

Electronic publication
