# Supplementary material for: Splicing factor TRA2B enhances synthesis of androgen receptor variant AR-V7 in prostate cancer cells
Source: J Clin Invest. 2026 Apr 1;136(7):e198264. doi: 10.1172/JCI198264 (PMC13038199; doi:10.1172/JCI198264)
Supplement: Supplemental data [file jci-136-198264-s221.pdf]

Supplementary information to accompany Brittain *et al.*, 'Splicing factor TRA2B enhances synthesis of androgen receptor variant AR-V7 in prostate cancer cells'

Contents

	Page number
Supplementary Figures S1-S14	2-15
Supplementary Tables S1-S11	See accompanying Excel file

Supplementary Figures

Supplementary Figure S1

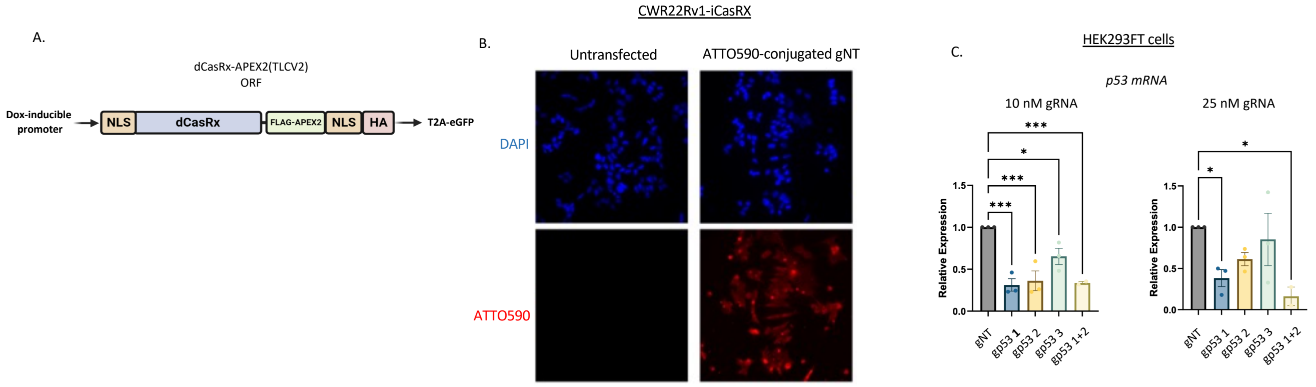

Supplementary Figure S1. Validating cellular mRNA knockdown capacity of CasRX. A. Visual summary of dCasRx-APEX2 ORF. N and C-terminal SV40 NLS flank the dCasRx-APEX2 ORF, which also contains a C-terminal HA tag. **B.** CWR22Rv1-iCasRx cells were transfected with 25 nM of a non-targeting gRNA (gNT) conjugated with a 3’ ATTO 590 fluorophore for 72-hours before cell fixation and imaged by fluorescence microscopy at 40x magnification. **C.** HEK293FT were transfected with a CasRx expression plasmid and incubated for 48 hours prior to transfection with synthetic gRNA oligos targeting p53 (gp53 1-3; gp53 1+2) or non-targeting (NT) gRNA (gNT) for a further 48 hours. RT-qPCR was subsequently used to assess knockdown efficacy of p53 transcript. qPCR data comprises n=3 independent biological replicates, plotted as mean ± SEM and subject to a one-way ANOVA (* = $p < 0.05$, *** = $p < 0.001$).

Supplementary Figure S2

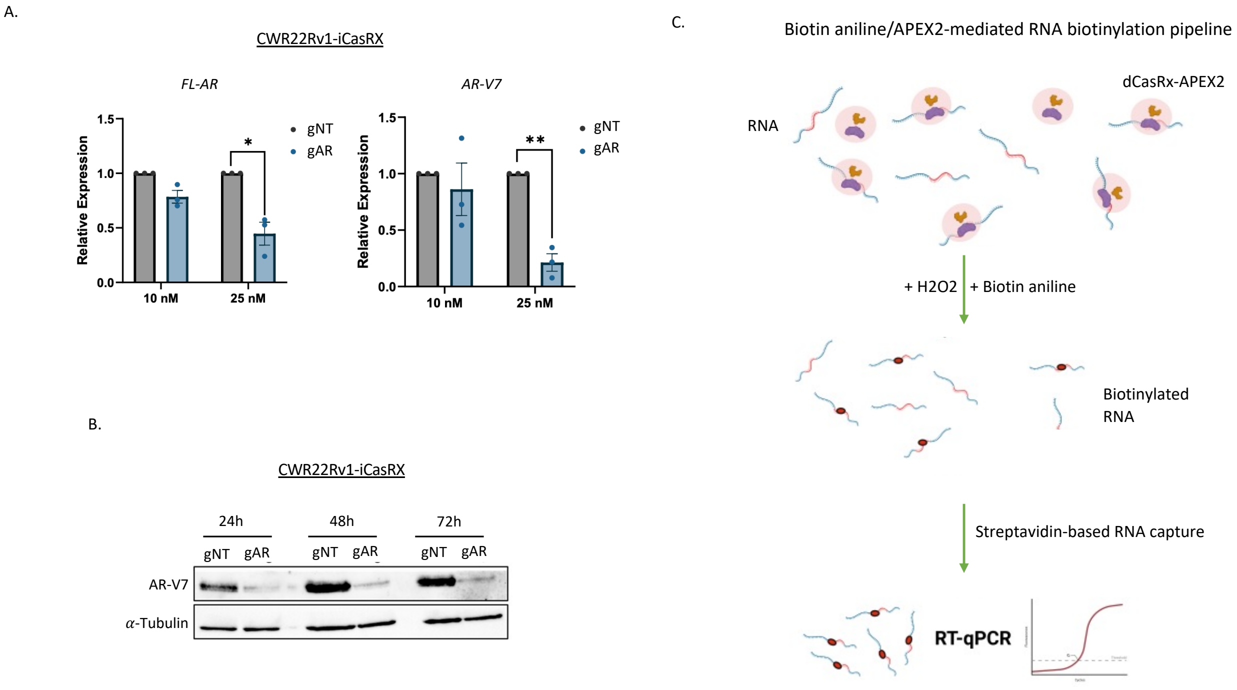

Supplementary Figure S2. CE3-targeting gRNA downregulates AR isoforms in CWR22Rv1 cells. A. CWR22Rv1-iCasRx were transfected with 10 nM or 25 nM of either gNT or gAR and induced with 1 μg/ml doxycycline for 72 hours prior to RT-qPCR to assess levels of *AR-FL* and *AR-V7* transcripts. Data comprises n=3 independent biological replicates, plotted as mean ± SEM and subject to an unpaired t-test (* = $p < 0.05$, ** = $p < 0.01$). **B.** CWR22Rv1-CasRx were transfected with 25 nM gNT or gAR and treated with 1 μg/ml doxycycline and harvested for western analysis using anti-AR-V7 and –α-tubulin antibodies 24, 48 and 72-hours later. **C.** Pipeline for dCasRx-APEX2-mediated RNA biotinylation. dCasRx-APEX2-gAR complexes selectively interact and biotinylate target RNAs in the presence of biotin aniline and H_2O_2 which are then captured by streptavidin-based immunoprecipitation and subject to RT-qPCR.

Supplementary Figure S3

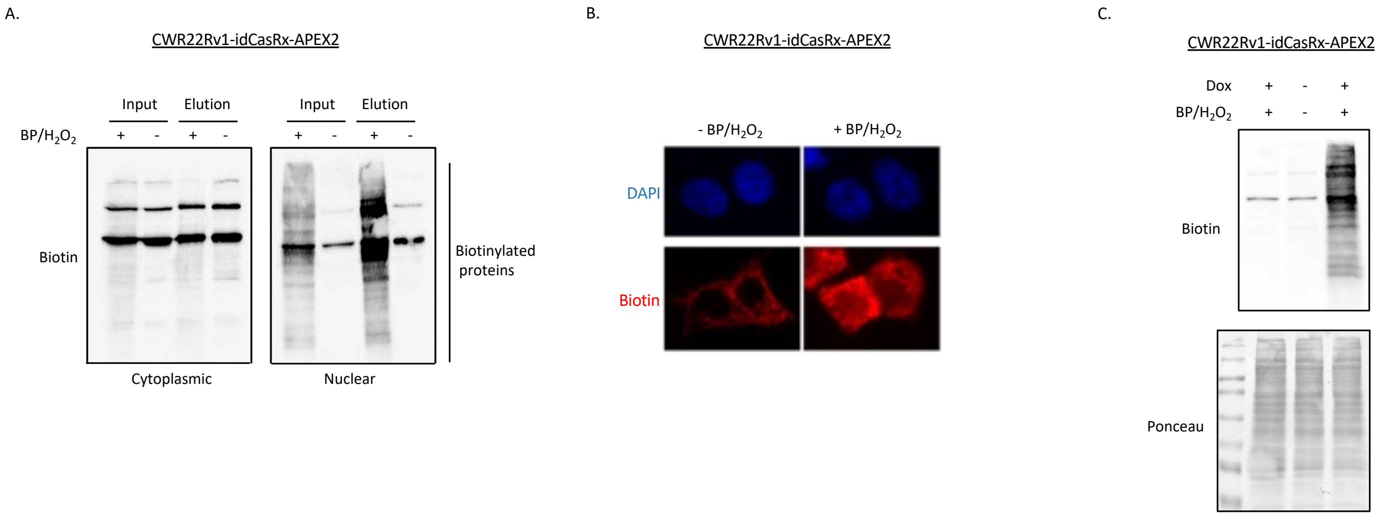

Supplementary Figure S3. Optimising dCasRX-APEX2-mediated proximal protein biotinylation in CWR22Rv1-idCasRX-APEX2 cells. A. CWR22Rv1-idCasRx-APEX2 were induced with 1 µg/ml doxycycline for 72 hours before treatment with +/- biotin phenol (BP) and H_2O_2 for 2 hours and 2 minutes, respectively, to activate proximal protein biotinylation. Cells were then subject to cytoplasmic-nuclear fractionation before biotinylated protein enrichment with streptavidin and elution. Western blot analysis of biotin signal in input and eluted fractions of each subcellular compartment was performed. **B.** CWR22Rv1-idCasRx-APEX2 were induced with 1 µg/ml doxycycline for 72 hours before proximity biotinylation was performed with BP and H_2O_2 for 2 hours and 2 minutes, respectively. Cells were then fixed, permeabilised and analysed by DAPI staining and anti-biotin immunofluorescence at 40x magnification. **C.** CWR22Rv1-idCasRx-APEX2 were treated with +/- 1 µg/ml doxycycline and BP/H_2O_2, before nuclear extraction and biotinylated protein content analysis by anti-biotin western blotting. Ponceau was used to confirm protein loading.

Supplementary Figure S4

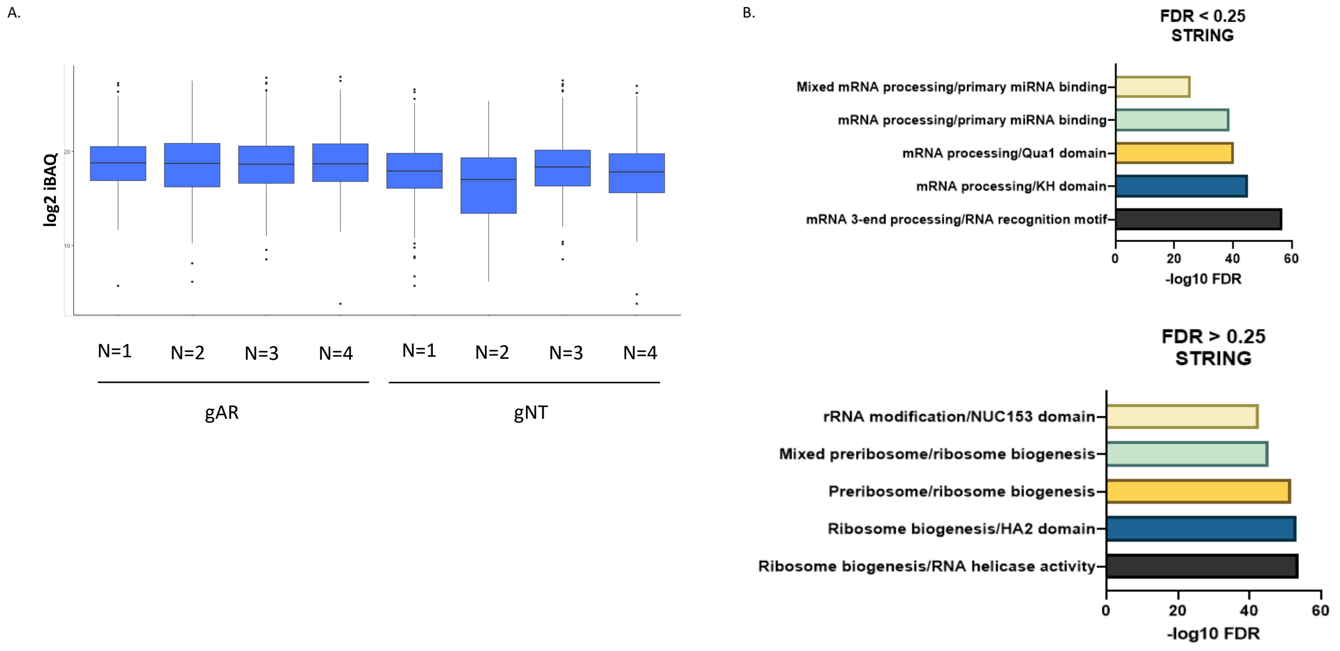

Supplementary Figure S4. Ensuring analysis of equal protein quantities between experimental arms enhances distinction of CE3-specific protein activities. A. Boxplot of log2 iBAQ values in AR g2 and NT experimental arms, subsequently used as input for *limma* analysis. Boxplots are N=1-4 for each indicated gRNA arm. **B.** KEGG analysis, performed using STRING, was applied to proteins in two separate groups representing those enriched by gAR at a *limma* FDR < 0.25 and > 0.25. The top 5 most enriched STRING network category terms are displayed for each group, ranked by -log10 STRING FDR.

Supplementary Figure S5

A.

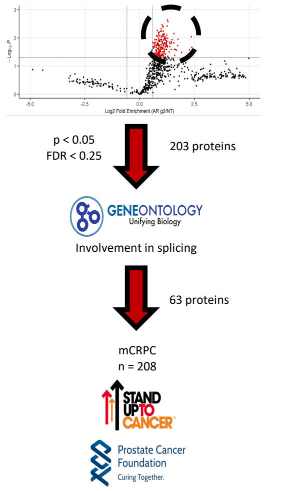

C.

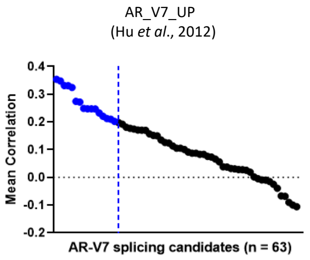

AR_V7_UP Q4 correlation

EIF4A3	TRA2B	DHX9	EFTUD2
SNRPD1	SRSF7	SNRPA	SNW1
SNRPD2	CDC5L	SF3B3	SNRPF
KHSRP	U2AF2	SF3B6	SNRNP200

D.

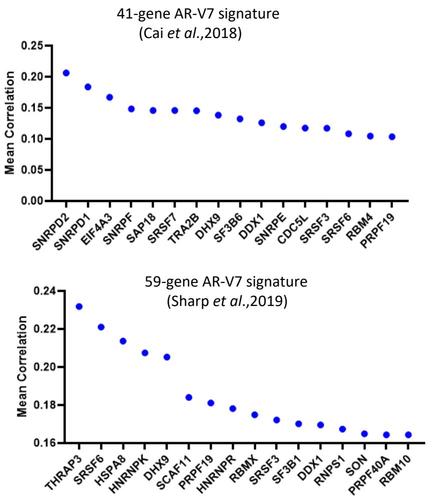

B.

Correlation with AR-V7 SRPM (Abida et al., 2019)
P< 0.05

SCAF11	SF3B6	HNRNPF	SON
RNPS1	U2AF2	SNRPF	EFTUD2
THRAP3	SYNCRIP	HNRNPK	DHX9
PRPF19	SRSF9	TAF15	CDC5L
RBM10	SRSF6	PRPF40A	TRA2B
KHSRP	HNRNPR	SFPQ	
SNRPD1	ESRP1	EIFA3	

E.

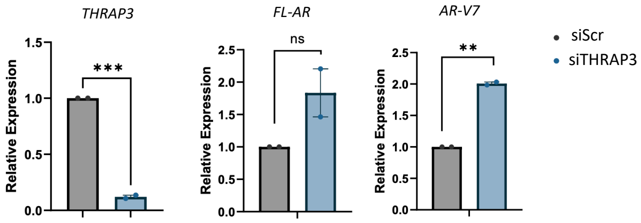

Supplementary Figure S5. Triangulation of proteomics and in silico data to identify potential spicing regulators of AR-V-encoding transcripts. A. Workflows to interrogate the 203 dCasRX-APEX CE3-interacting proteins at p < 0.05 and FDR < 0.25. This panel was further refined for proteins with established splicing activity, yielding a list of 63. Two large RNA-Seq datasets obtained from PCa (TCGA, n=320) and mCRPC (SU2C/PCF, n=208) patients were analysed for associations between *AR-V7* splicing, activity signatures and expression of genes encoding the panel of 63 proteins. **B.** Genes encoding this list of 63 proteins were examined for correlation between gene expression (FPKM) and *AR-V7* splicing (SRPM)(SU2C/PCF dataset), the latter being classed as the number of RNA-Seq mapped reads spanning the exon 3/CE3 boundary per million reads. Expression of 26 genes correlated with AR-V7 SRPM at p < 0.05 using Pearson's correlation coefficient. **C.** Mean Pearson's correlation analysis was performed for each of the 63-gene list with the *AR_V7_UP* 25-gene signature (10); Q4 - top quartile marked by blue dotted line and points). Fisher z-transformation was applied for calculation of mean correlation across the 25-gene signature. Q4 correlating genes are displayed in the adjoining table. **D.** Correlation analysis of genes encoding the 63-protein list of splicing regulators against an AR-V7 target gene signatures (11,12) confirmed to associate with AR-V7 expression in CRPC tumour cohorts. Mean Pearson's coefficient is plotted for the top quartile of correlated genes. **E.** CWR22Rv1 transfected with either scrambled (siScr) or THRAP3 (siTHRAP3) siRNAs for 72 hours were subject to RT-qPCR to analyse *THRAP3*, *FL-AR* and *AR-V7* transcript. Data comprises n = 2 independent biological replicates, plotted as mean ± SEM. Unpaired t-test was used for determination of statistical significance (* = p < 0.05, ** = p < 0.01, ns = p > 0.05).

Supplementary Figure S6

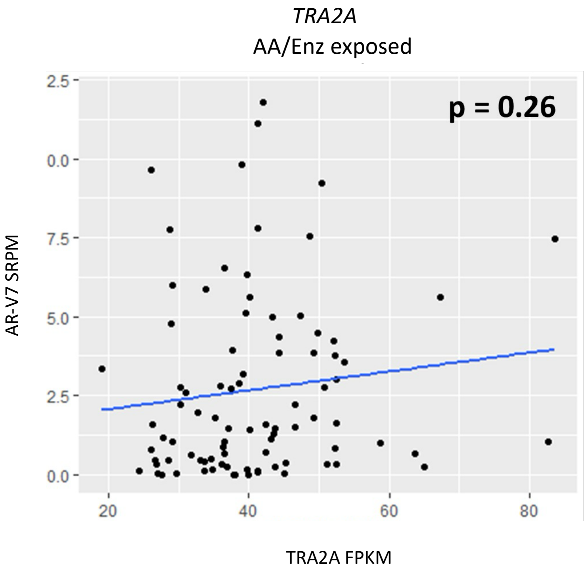

Supplementary Figure S6. TRA2A expression does not correlate with *AR-V7* transcripts. A. Mean *AR-V7* SRPM was compared between AA/ENZ naïve and exposed patients from the SU2C/PCF mCRPC cohort (13). AA/ENZ naïve comprised n = 106 patients, AA/ENZ exposed comprised n = 89 patients. *TRA2A* FPKM expression was correlated with *AR-V7* SRPM in AA/ENZ naïve and exposed patients.

Supplementary Figure S7

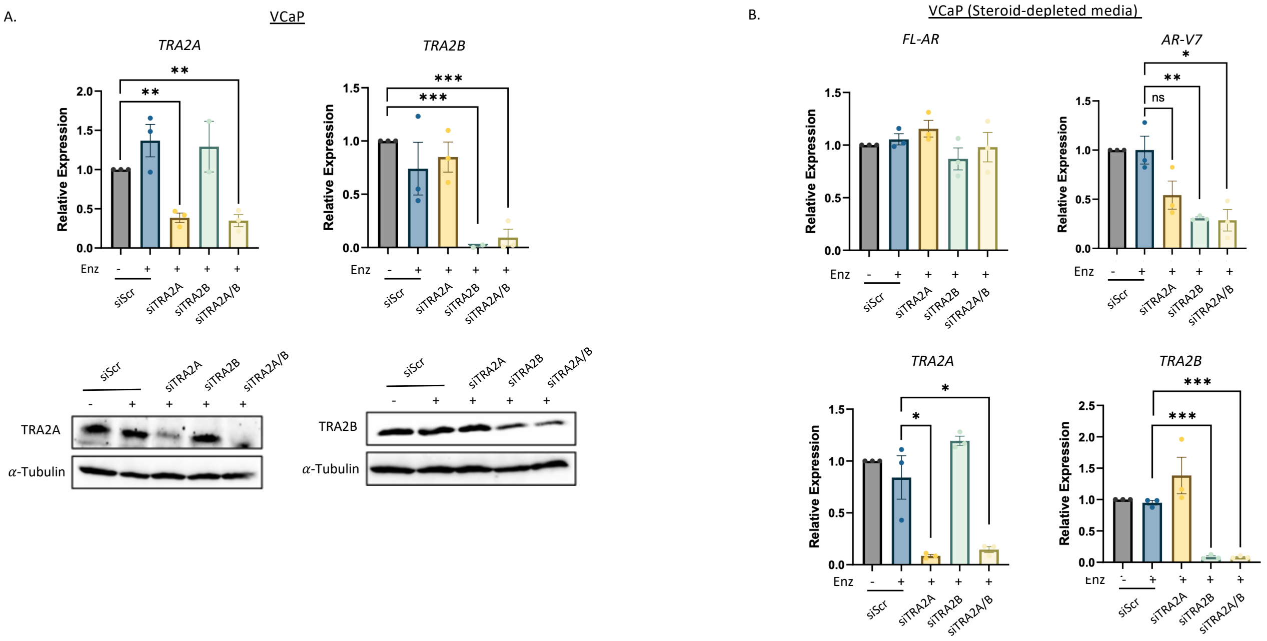

Supplementary Figure S7. TRA2B and its paralogue TRA2A regulate *AR-V7* synthesis in VCaP cells. A. VCaP cells grown in serum-containing media were transfected with either TRA2A, TRA2B or both siRNAs and treated +/- 10 µM enzalutamide (Enz) for 72 hours before RT-qPCR and western analysis of AR-FL and AR-V7 transcript and protein levels. **B.** VCaP cells grown in steroid-depleted media were transfected as in (A) and subject to RT-qPCR to assess AR isoform and *TRA2A* and *TRA2B* transcript levels. All qPCR data comprises n=3 independent biological replicates, plotted as mean ± SEM and subject to a one-way ANOVA (* = $p < 0.05$, ** = $p < 0.01$, *** = $p < 0.001$, ns = $p > 0.05$).

Supplementary Figure S8

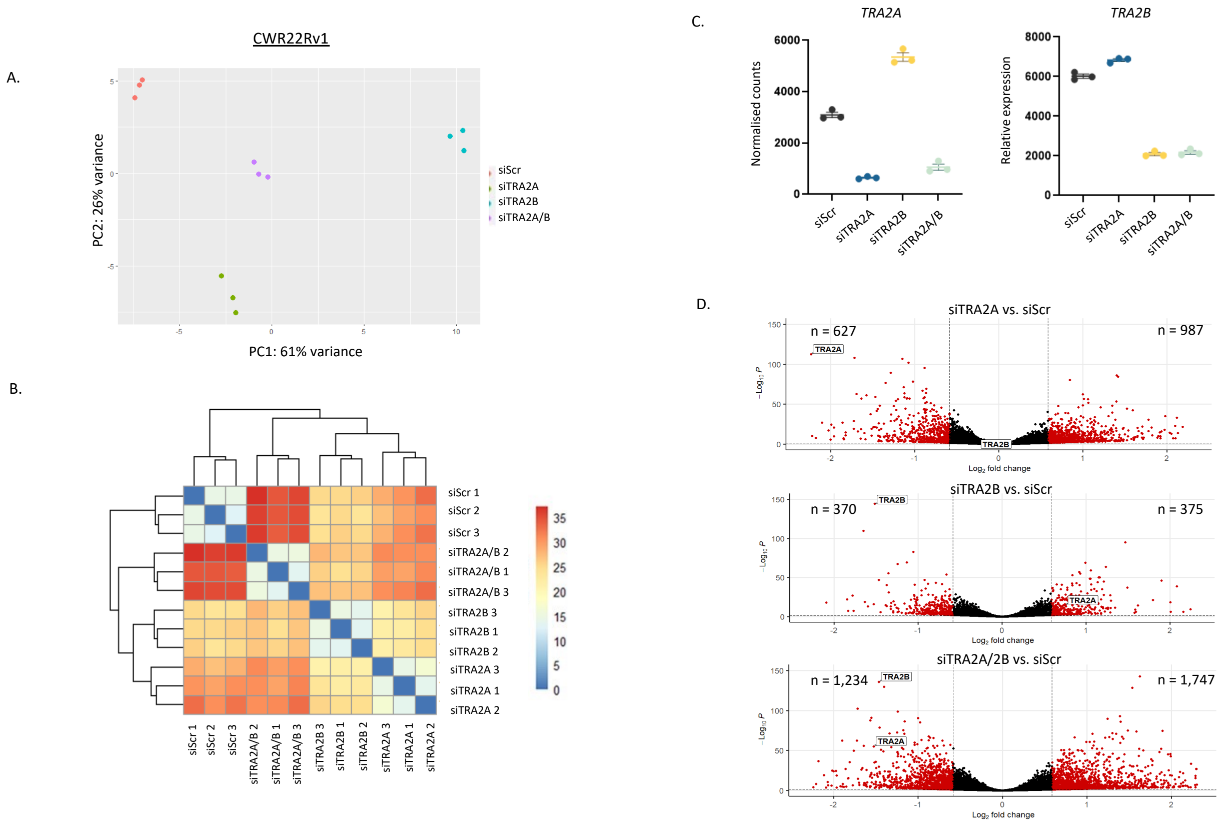

Supplementary Figure S8. Validating RNA-sequencing data using principal component analysis and hierarchical clustering. A. Principal component analysis (PCA) of *DESeq2*-normalised gene counts. **B.** Heatmap of hierarchical clustering determined by Euclidean distance between normalised gene counts for each sample. Colour scale denotes count profile divergence between sample pairings from perfect overlap (blue) to maximum divergence (red). **C.** Summary of RNA-Seq gene expression counts, normalised by *DESeq2*, for *TRA2A* and *TRA2B* in each sample. **D.** Volcano plot of DEGs resulting from each respective TRA2 siRNA vs NT DGEA. *TRA2A* and *TRA2B* are highlighted in boxes. Cutoffs for significantly DEGs (points in red) are FDR < 0.05 and linear fold change +/- 1.5.

Supplementary Figure S9

A.

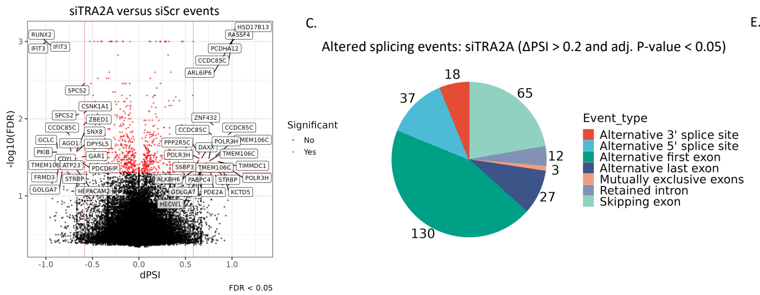

B.

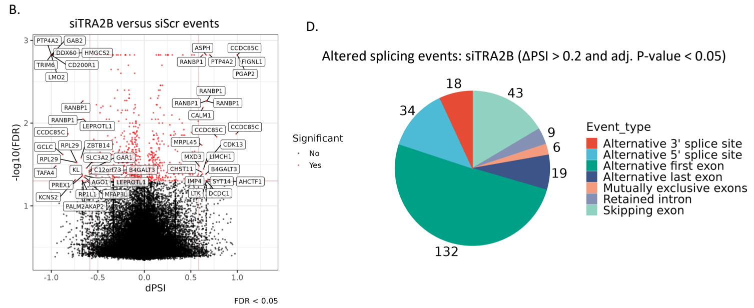

E.

siTRA2A/siTRA2B versus siScr events

Index	Name	P-value	Adjusted p-value	Odds Ratio	Combined score
1	Metal Ion Binding (GO:0046872)	0.002109	0.1752	3.77	23.24
2	mRNA Binding (GO:0003729)	0.002615	0.1752	4.66	27.72
3	Kinase Inhibitor Activity (GO:0019210)	0.01241	0.2176	12.69	55.71
4	Phosphatidylinositol Binding (GO:0035091)	0.01274	0.2176	6.52	28.44
5	Calcium Ion Binding (GO:0005509)	0.01485	0.2176	3.66	15.39
6	Protein Serine/Threonine Kinase Inhibitor Activity (GO:0030291)	0.01785	0.2176	10.38	41.79
7	3-hydroxyacyl-CoA Dehydrogenase Activity (GO:0003857)	0.02205	0.2176	56.55	215.71
8	lncRNA Binding (GO:0106222)	0.02205	0.2176	56.55	215.71
9	Dihydropyrimidinase Activity (GO:0004157)	0.02205	0.2176	56.55	215.71
10	Double-Stranded Methylated DNA Binding (GO:0010385)	0.02205	0.2176	56.55	215.71

F.

siTRA2A versus siScr events

Index	Name	P-value	Adjusted p-value	Odds Ratio	Combined score
1	Single-Stranded RNA Binding (GO:0003727)	0.002809	0.1258	28.08	164.96
2	Double-Stranded RNA Binding (GO:0003725)	0.006854	0.1258	17.48	87.08
3	Dihydropyrimidinase Activity (GO:0004157)	0.008720	0.1258	146.77	696.01
4	Acid-Amino Acid Ligase Activity (GO:0016881)	0.01046	0.1258	117.41	535.47
5	Phosphate Ion Binding (GO:0042301)	0.01564	0.1258	73.37	305.06
6	cAMP-dependent Protein Kinase Inhibitor Activity (GO:0004862)	0.01564	0.1258	73.37	305.06
7	cGMP Binding (GO:0030553)	0.01737	0.1258	65.22	264.33
8	cAMP-dependent Protein Kinase Regulator Activity (GO:0008603)	0.01909	0.1258	58.69	232.34
9	Hydrolase Activity, Acting on Carbon-Nitrogen (But Not Peptide) Bonds, in Cyclic Amides (GO:0016812)	0.02080	0.1258	53.35	206.61
10	SUMO Ligase Activity (GO:0061665)	0.02252	0.1258	48.90	185.51

G.

siTRA2B versus siScr events

Index	Name	P-value	Adjusted p-value	Odds Ratio	Combined score
1	Double-Stranded RNA Binding (GO:0003725)	0.0003575	0.02860	24.38	193.48
2	snoRNA Binding (GO:0030515)	0.001662	0.06650	37.15	237.75
3	Single-Stranded RNA Binding (GO:0003727)	0.003479	0.09277	25.04	141.74
4	L-leucine Transmembrane Transporter Activity (GO:0015190)	0.009713	0.1114	131.30	608.47
5	Acid-Amino Acid Ligase Activity (GO:0016881)	0.01164	0.1114	105.03	467.70
6	Glucosidase Activity (GO:0015926)	0.01164	0.1114	105.03	467.70
7	L-alanine Transmembrane Transporter Activity (GO:0015180)	0.01550	0.1114	75.02	312.60
8	Adenylate Cyclase Regulator Activity (GO:0010854)	0.01550	0.1114	75.02	312.60
9	RNA Polymerase II CTD Heptapeptide Repeat Kinase Activity (GO:0008353)	0.01550	0.1114	75.02	312.60
10	Branched-Chain Amino Acid Transmembrane Transporter Activity (GO:0015658)	0.01742	0.1114	65.63	265.84

Supplementary Figure S9. Global splicing analysis of individual TRA2A and TRA2B knockdowns in PC cells. RNA sequencing data derived from CWR22Rv1 cells depleted of TRA2A **(A)** and TRA2B **(B)** was subject to SUPPA2 analysis to assess differential splicing events that passed cutoffs of ΔPSI ± 0.6 and FDR <0.05, and are shown as Volcano plots annotated with significantly altered genes IDs shown in red. Global splicing patterns of CWR22Rv1 cells depleted of TRA2A **(C)** and TRA2B **(D)** were quantified by category (e.g., alternative first exon and skipping exon). Events that passed a significant Δproportion spliced in (ΔPSI) value ± 0.2 (*P* value of <0.05) were plotted as a pie-chart including event quantification. Significantly altered splicing events (ΔPSI and *P* value) in unique genes in response to depletion of TRA2A/TRA2B **(E)**, TRA2A **(F)** and TRA2B **(G)** were run through Enrichr using molecular function gene ontology (GO) terms filters to identify involvement in cellular functions.

Supplementary Figure S10

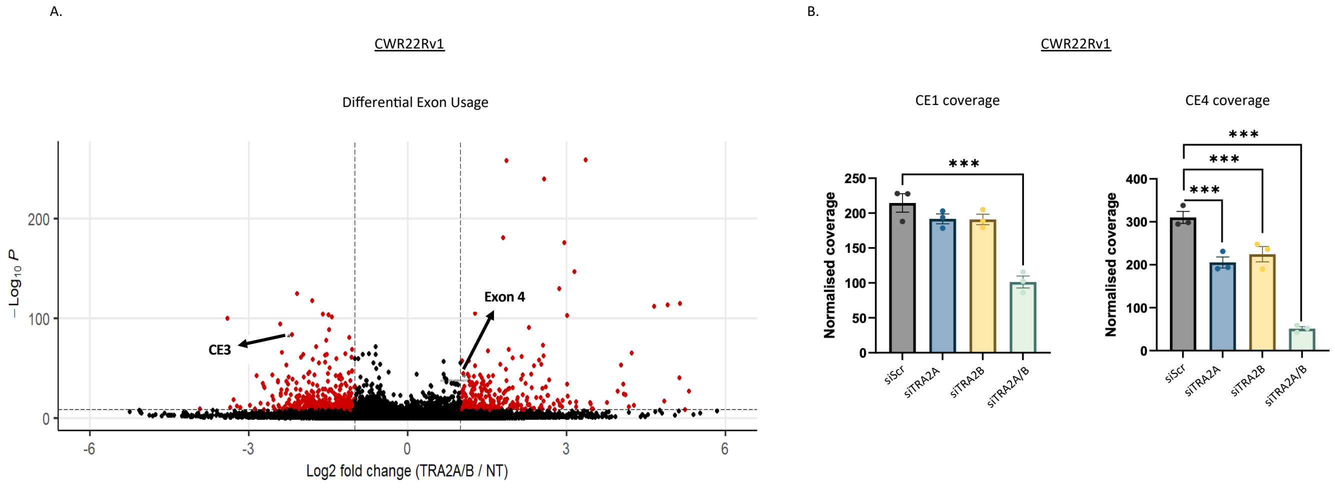

Supplementary Figure S10. Combined TRA2A and TRA2B depletion increases exon 4 inclusion at the expense of cryptic exons encoding AR-Vs. A. Volcano plot illustrates -log10 FDR (y-axis) vs log2 fold change (x-axis) of differential exon usage in TRA2A/B vs NT-transfected samples, as calculated by *JunctionSeq*. Only exons differentially used between samples at an FDR < 0.01 are included. Exons highlighted in red meet a cut-off of linear fold change +/- 2 and FDR < 0.00000001. *AR* CE3 and exon 4 are highlighted. **B.** *JunctionSeq* differential exon usage analysis results for cryptic exons CE1 and CE4 which encode AR-Vs including, AR-V1 and AR-V3, are shown as normalised exon counts ± SEM for each sample. Significance values denote *JunctionSeq* FDR (*** = FDR < 0.00001). Only results significant at α 0.001 or lower have significance denoted.

Supplementary Figure S11

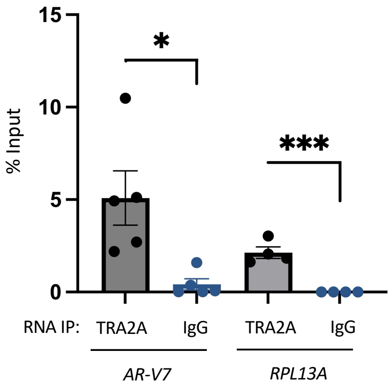

Supplementary Figure S11. TRA2A binds *AR-V7* transcript. CWR22Rv1 cells were subject to RNA immunoprecipitation using either anti-TRA2A or control antibodies prior to qRT-PCR to quantify % input of TRA2A interaction with AR-V7 and control RPL13A transcripts (* and *** represent p value < 0.05 and 0.001, respectively, as calculated using a paired T-test from at least three independent experiments).

Supplementary Figure S12

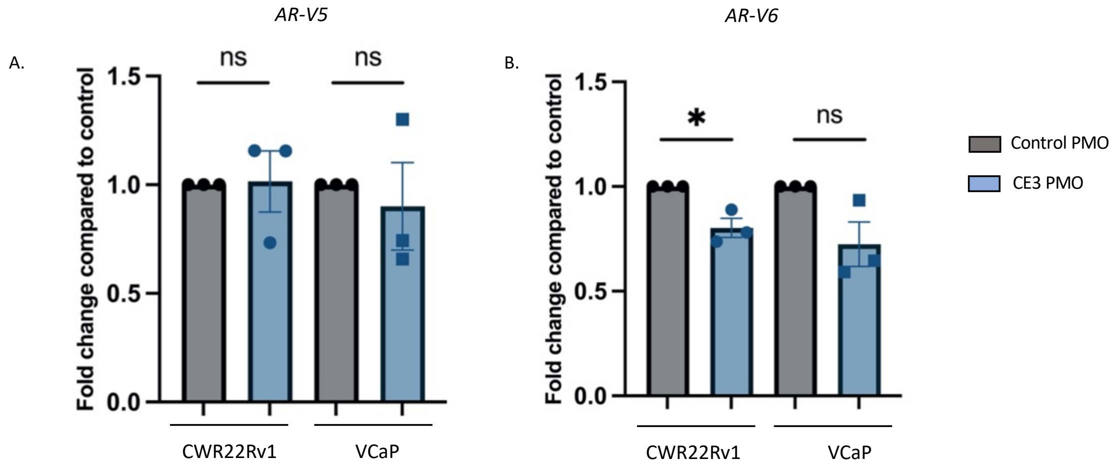

Supplementary Figure S12. AR-V5 and AR-V6 transcript abundance is not markedly impacted by a CE3-targeting morpholino. CWR22Rv1 cells were transfected with 10 µM CE3-targeting or control PMO for 48 hours prior to RT-qPCR analysis expression of AR-V5 **(A)** and AR-V6 **(B)**. qPCR data comprises n=3 independent biological replicates, plotted as mean ± SEM and subject to unpaired t-test (* = $p < 0.05$).

Supplementary Figure S13

CWR22Rv1

A.

41-gene AR-V7 signature
(Cai *et al.*,2018)

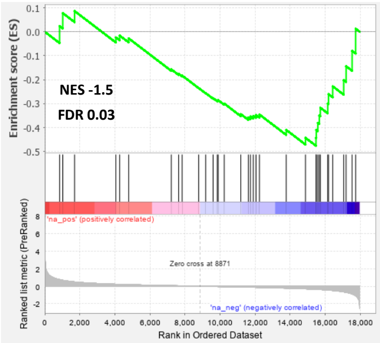

TRA2A/B KD

B.

Hallmark_*Androgen response*

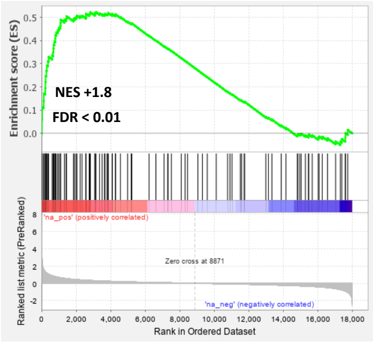

TRA2A/B KD

Supplementary Figure S13. Combined TRA2A and TRA2B depletion has opposing effects on AR-V7 and FL-AR signalling. Geneset enrichment analysis (GSEA) using **(A)** a published 41-gene AR-V7 expression signature (14) and **(B)** the Hallmark_Androgen response was performed on differentially-expressed genes identified in response to combined TRA2A and TRA2B knockdown in CWR22Rv1 cells.

Supplementary Figure S14

A.

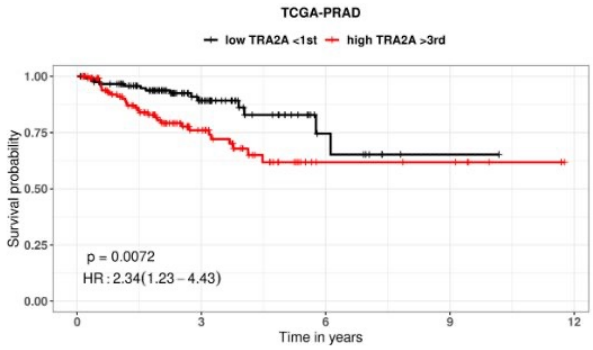

B.

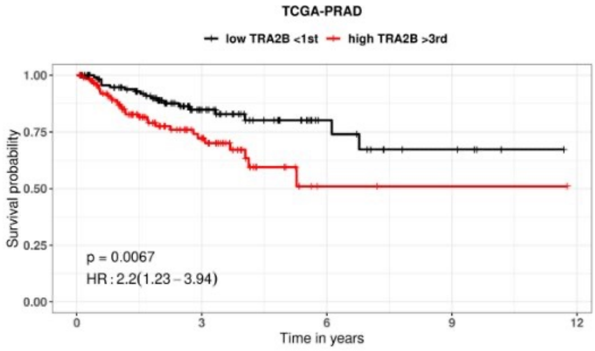

Supplementary Figure S14. Elevated TRA2 protein levels associate with reduced overall survival in TCGA PRAD cohort. Differences in progression-free interval times between the top (red) and bottom (black) quartiles of **(A)**_TRA2A_ and **(B)**_TRA2B_ expressing patients in the TCGA-PRAD cohort were determined by logrank test. Survival analysis in was performed using the Tumor online Prognostic analysis Platform (ToPP) (15).
