# Supplementary material for: Splicing factor TRA2B enhances synthesis of androgen receptor variant AR-V7 in prostate cancer cells
Source: J Clin Invest. 2026 Apr 1;136(7):e198264. doi: 10.1172/JCI198264 (PMC13038199; doi:10.1172/JCI198264)

Figure 1B

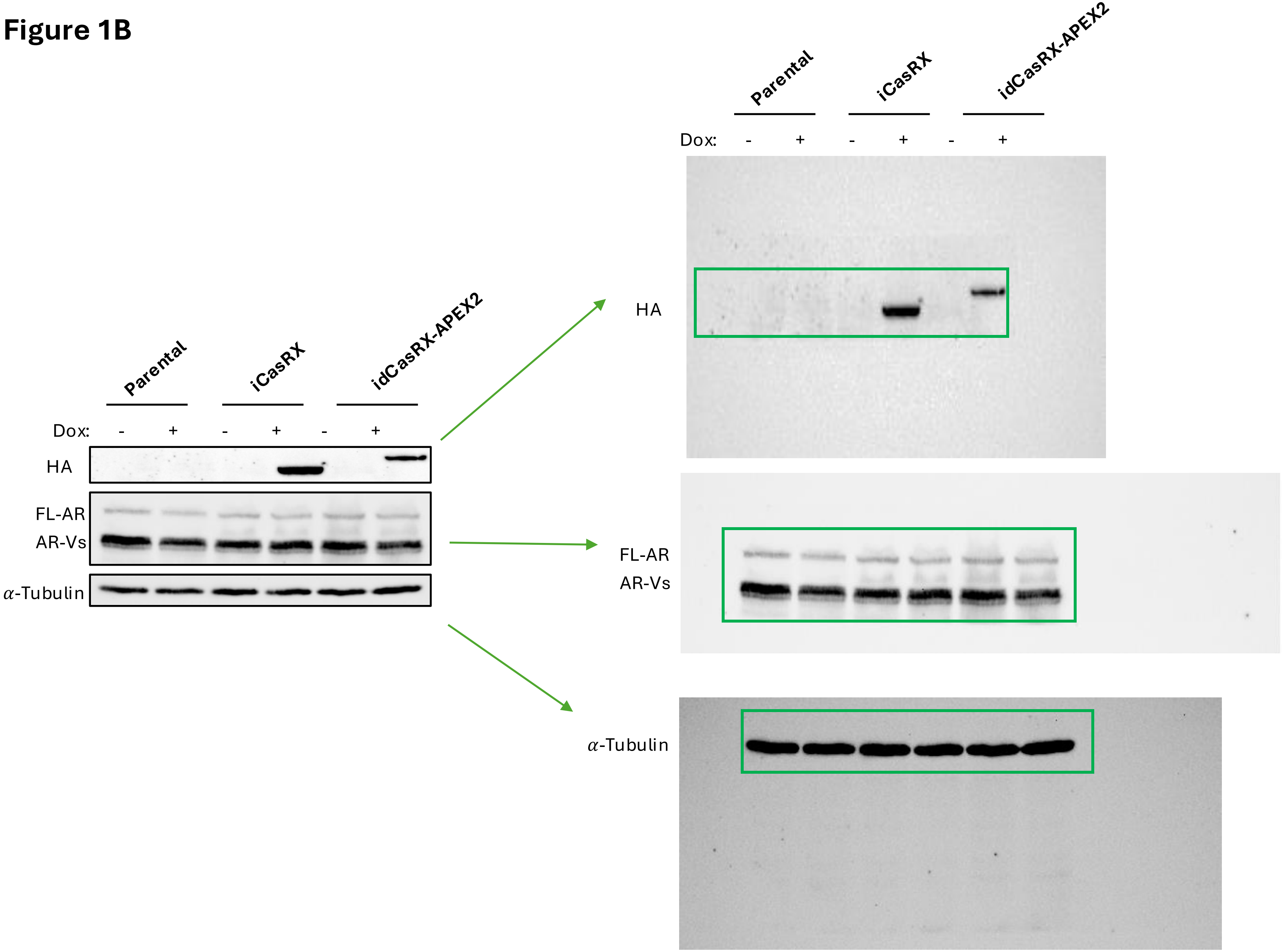
Parental
iCasRX
idCasRX-APEX2
Dox: - + - + - +
HA
FL-AR
AR-Vs
α-Tubulin
Parental
iCasRX
idCasRX-APEX2
Dox: - + - + - +
HA
FL-AR
AR-Vs
α-Tubulin

Figure 1F

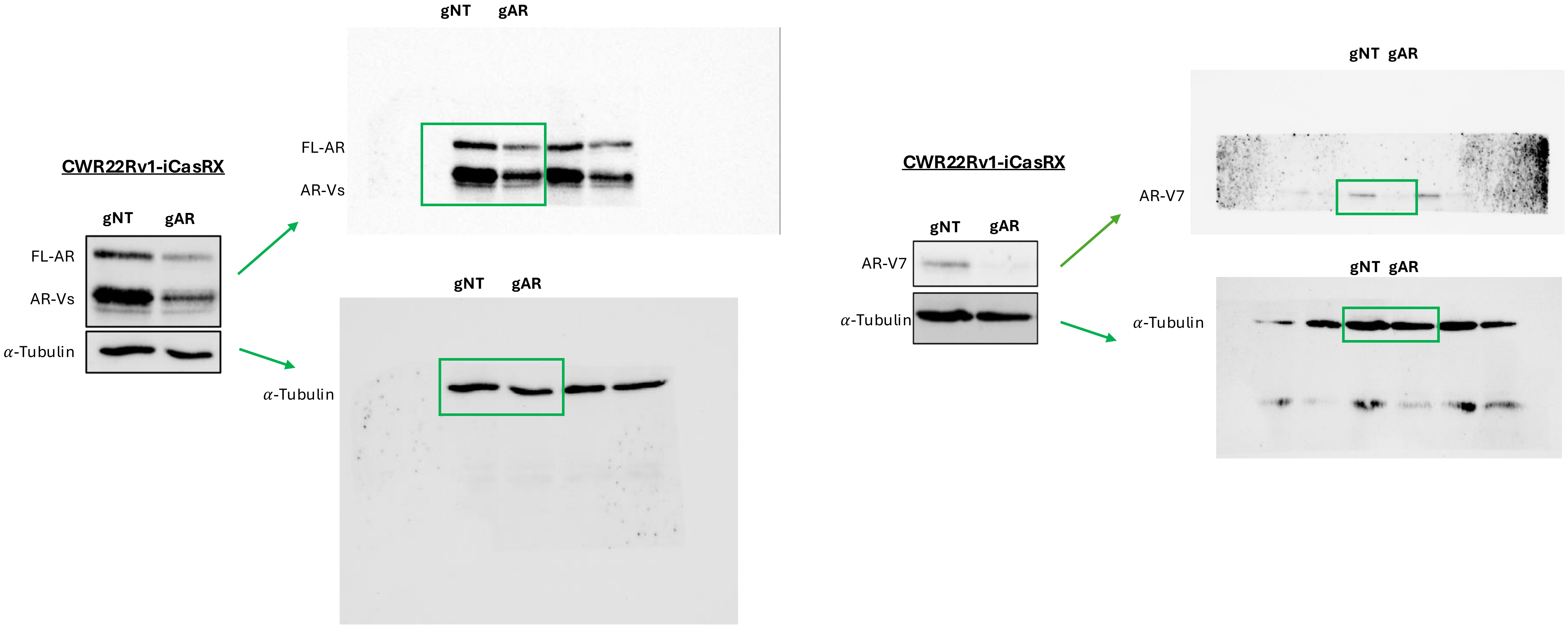
CWR22Rv1-iCasRX
gNT
gAR
FL-AR
AR-Vs
α-Tubulin
gNT
gAR
FL-AR
AR-Vs
gNT
gAR
α-Tubulin
CWR22Rv1-iCasRX
gNT
gAR
AR-V7
α-Tubulin
gNT
gAR
AR-V7
gNT
gAR
α-Tubulin

Figure 1G

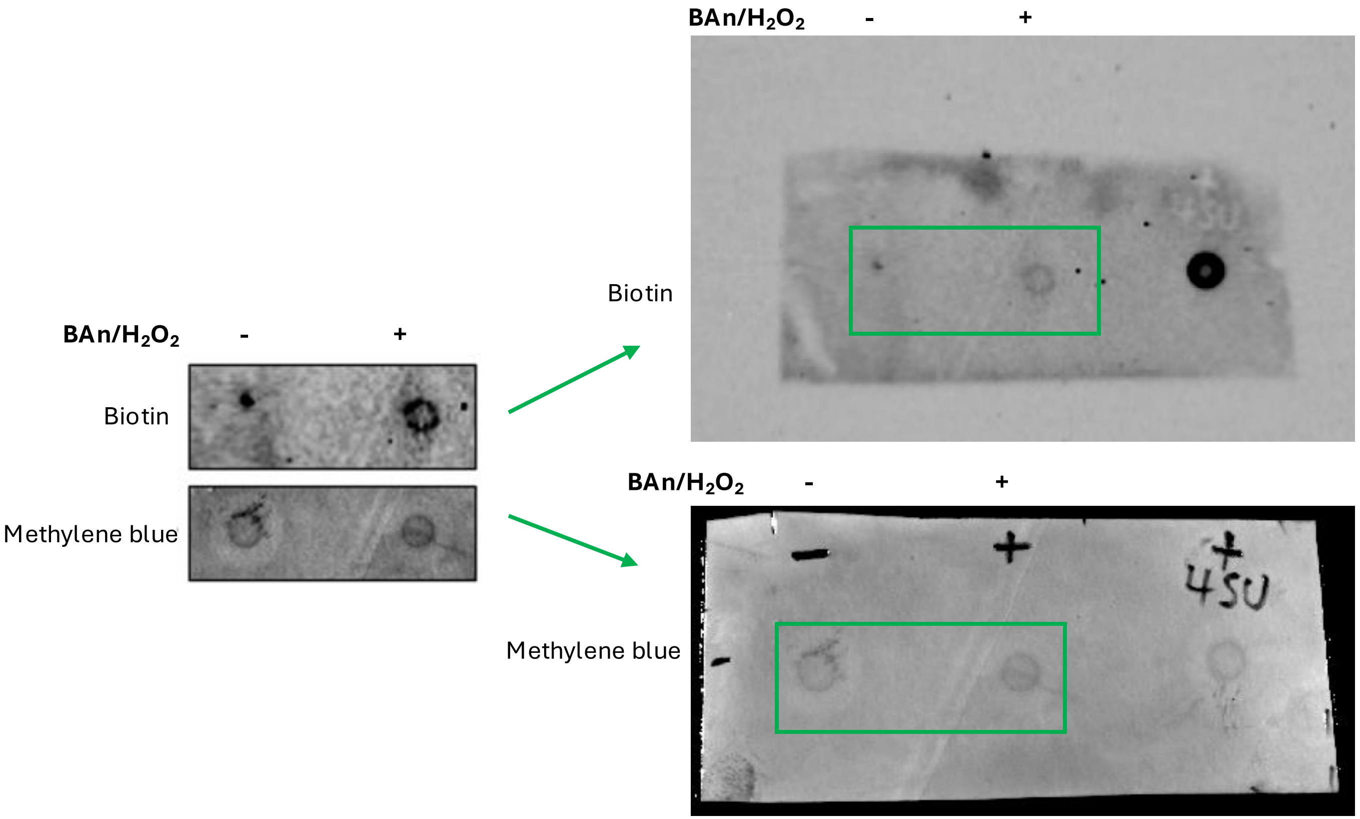
BAn/H_2O_2 - +
Biotin
Methylene blue
BAn/H_2O_2 - +
Biotin
BAn/H_2O_2 - +
Methylene blue
- + + 4SU

Figure 1I

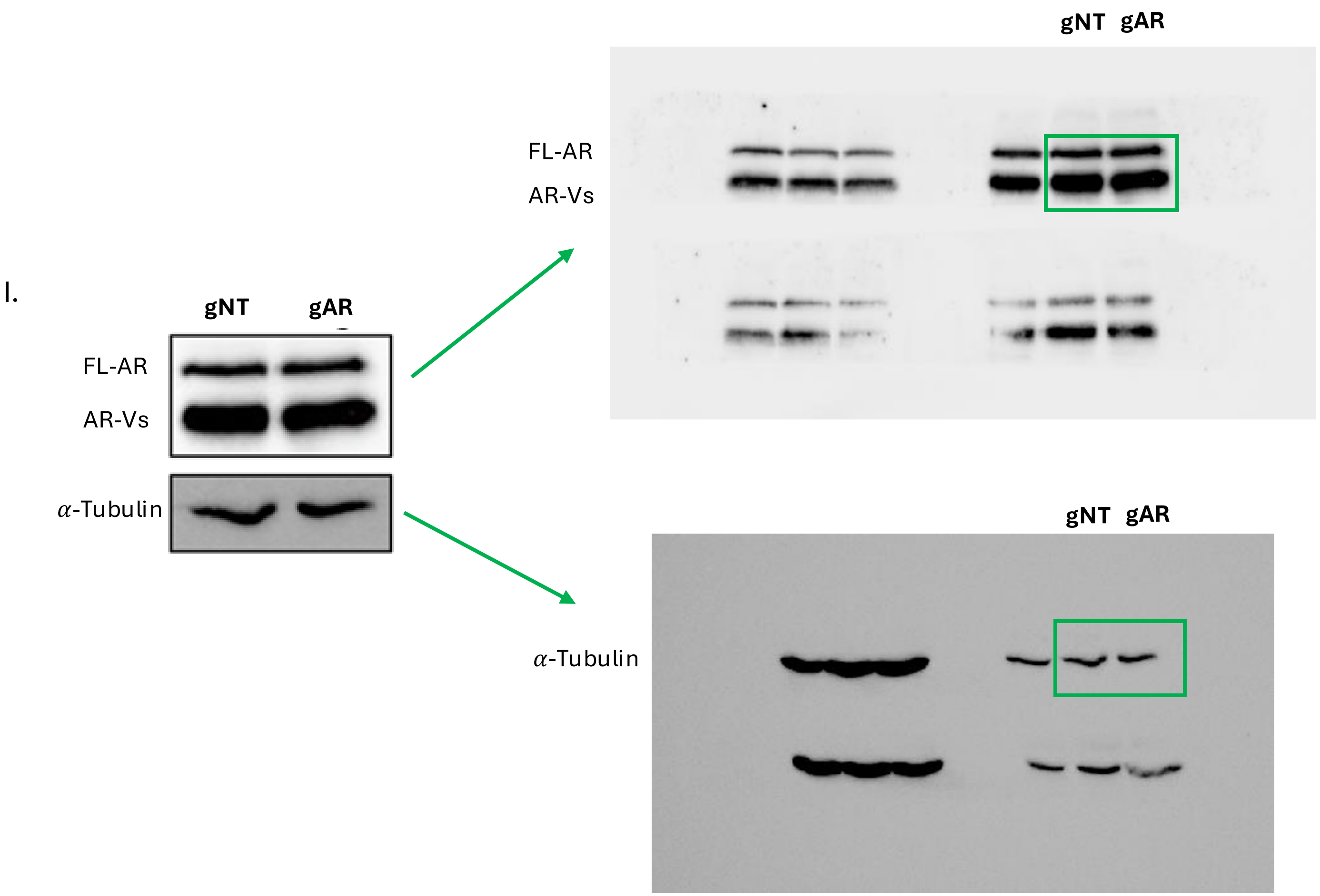

I.
gNT
gAR
FL-AR
AR-Vs
α-Tubulin
gNT gAR
FL-AR
AR-Vs
gNT gAR
α-Tubulin

Figure 3D

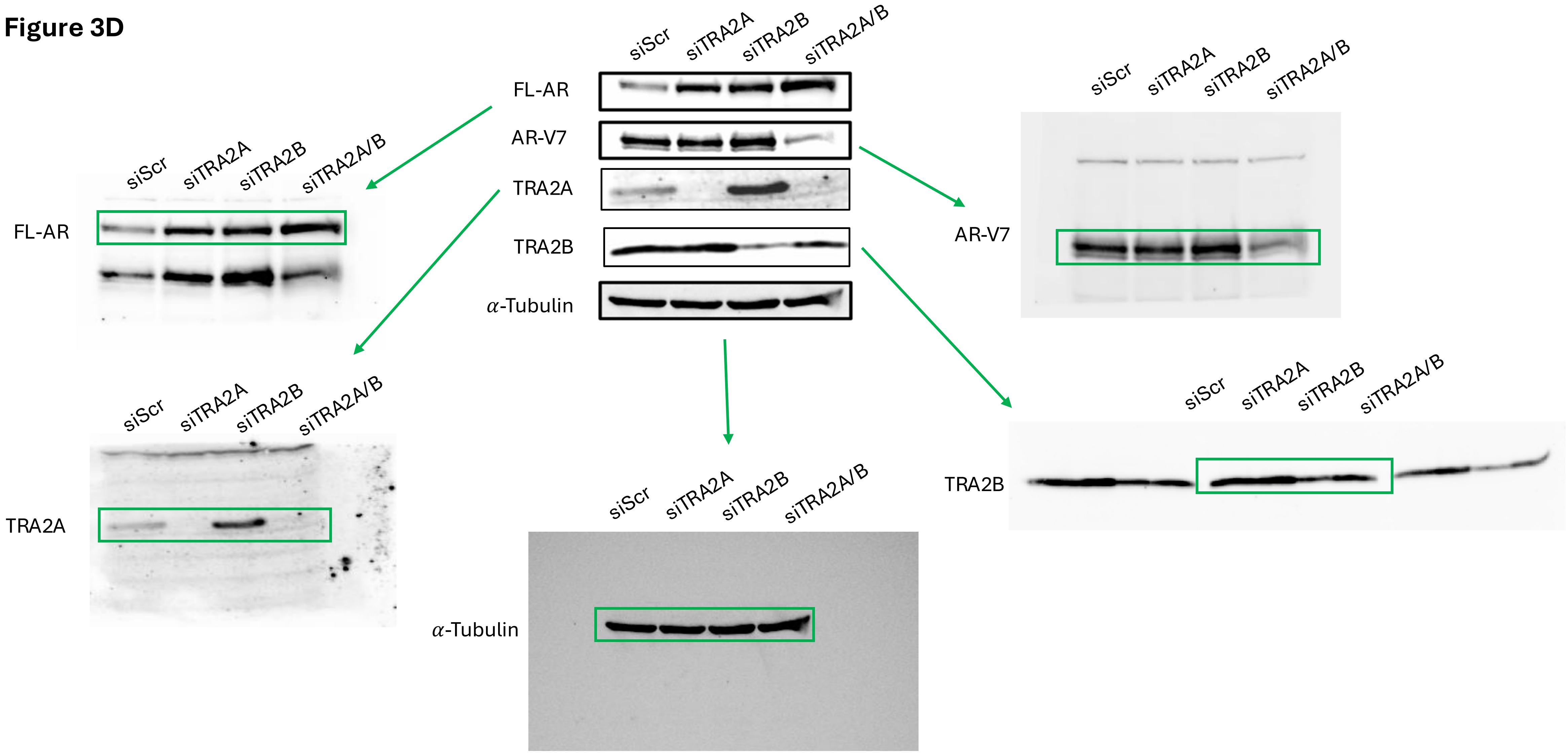

siScr
siTRA2A
siTRA2B
siTRA2A/B
FL-AR
AR-V7
TRA2A
TRA2B
α-Tubulin

Figure 3E

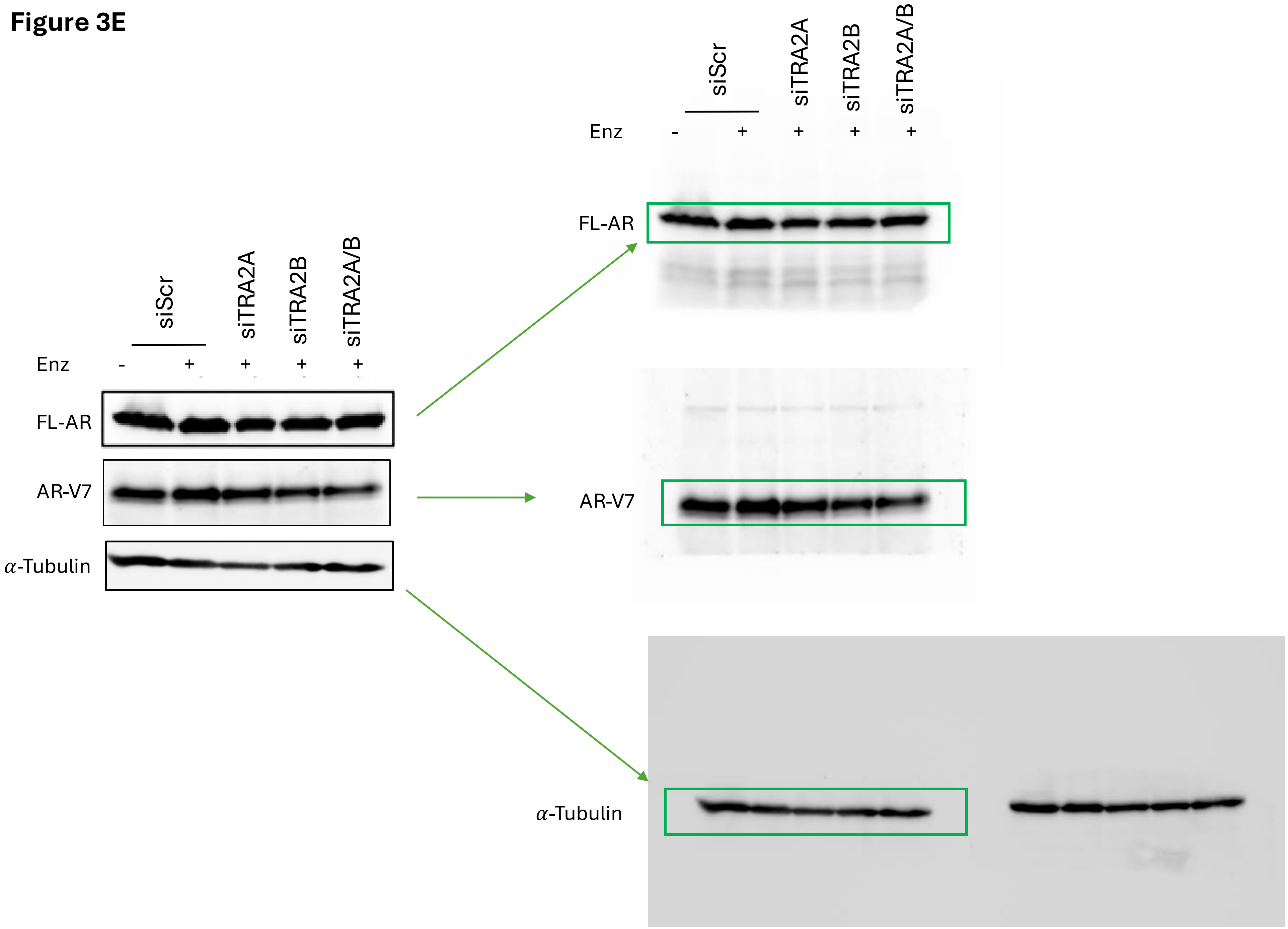
siScr
siTRA2A
siTRA2B
siTRA2A/B
Enz
- + + + +
FL-AR
AR-V7
α-Tubulin
siScr
siTRA2A
siTRA2B
siTRA2A/B
Enz
- + + + +
FL-AR
AR-V7
α-Tubulin

Supplementary Figure S2B

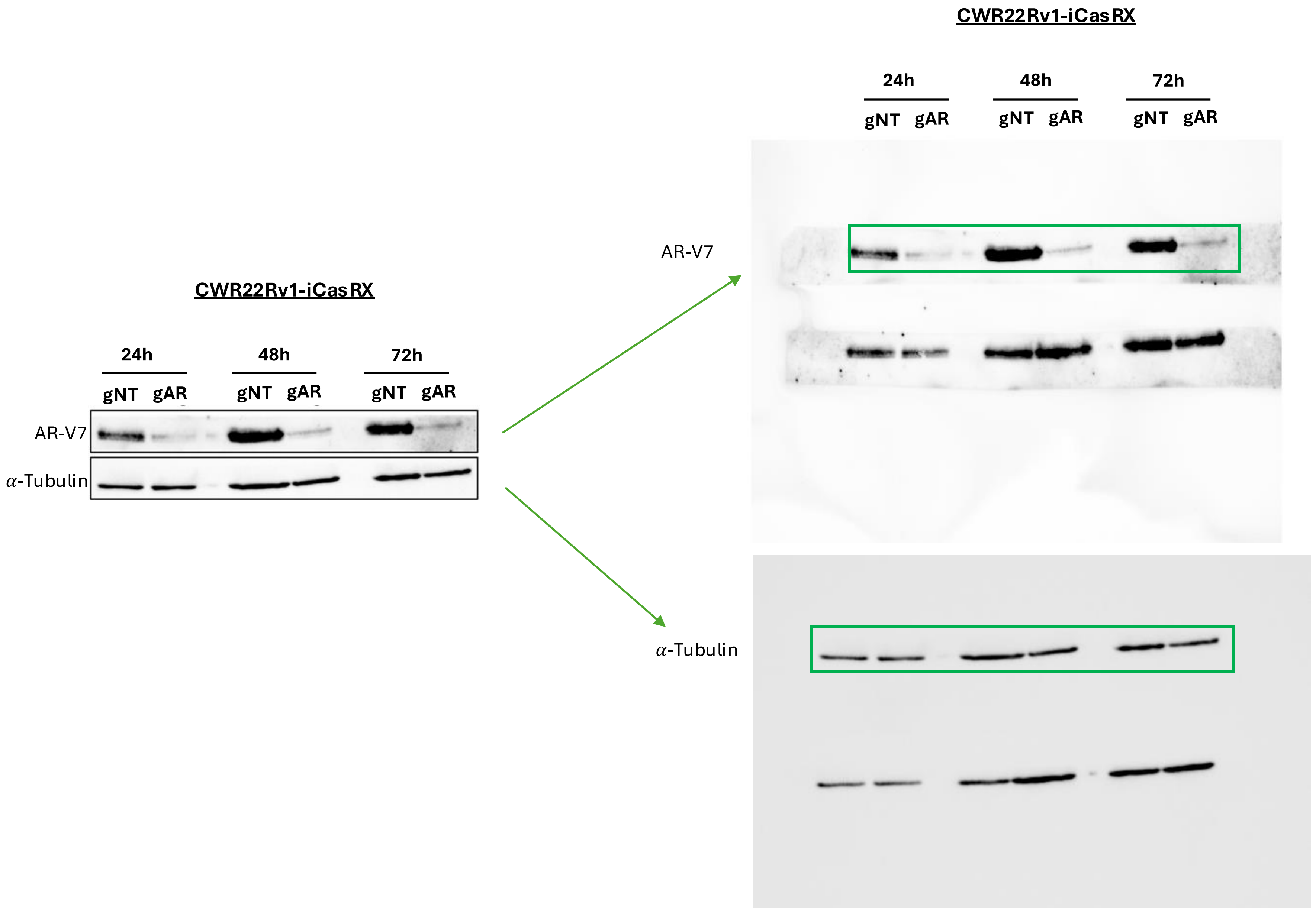

Supplementary Figure S7

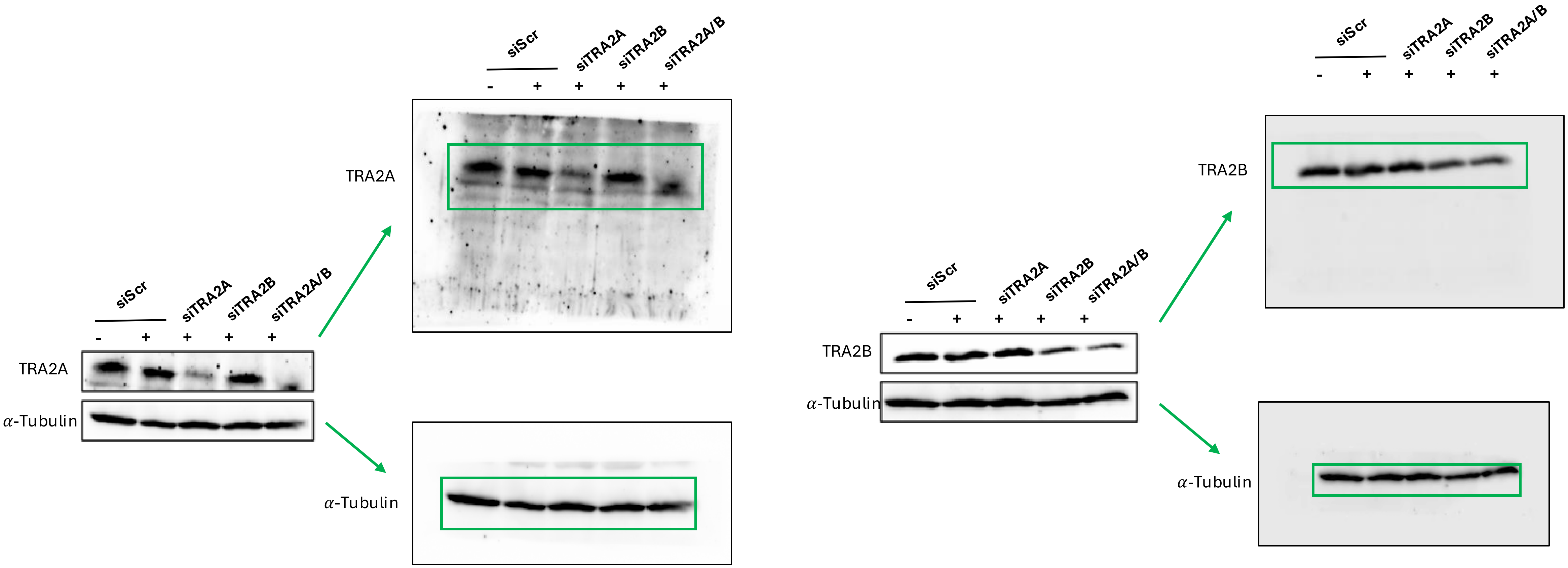

Supplement: Unedited blot and gel images [file jci-136-198264-s222.pdf]
